# *Escherichia coli* has robust regulatory mechanisms against elevated peptidoglycan cleavage by lytic transglycosylases

**DOI:** 10.1016/j.jbc.2023.104615

**Published:** 2023-03-16

**Authors:** Yaquan Liang, Yilin Zhao, Jeric Mun Chung Kwan, Yue Wang, Yuan Qiao

**Affiliations:** 1 School of Chemistry, Chemical Engineering and Biotechnology, Nanyang Technical University, Singapore; 2 A∗STAR Infectious Disease Labs, Agency for Science, Technology and Research, Singapore

**Keywords:** cell wall, peptidoglycan, Gram-negative bacteria, lytic transglycosylases, stress responses, antibiotics, muropeptides, analytic chemistry

## Abstract

Peptidoglycan (PG) is an essential and conserved exoskeletal component in all bacteria that protects cells from lysis. Gram-negative bacteria such as *Escherichia coli* encode multiple redundant lytic transglycosylases (LTs) that engage in PG cleavage, a potentially lethal activity requiring proper regulation to prevent autolysis. To elucidate the potential effects and cellular regulatory mechanisms of elevated LT activity, we individually cloned the periplasmic domains of two membrane-bound LTs, MltA and MltB, under the control of the arabinose-inducible system for overexpression in the periplasmic space in *E. coli*. Interestingly, upon induction, the culture undergoes an initial period of cell lysis followed by robust growth restoration. The LT-overexpressing *E. coli* exhibits altered morphology with larger spherical cells, which is in line with the weakening of the PG layer due to aberrant LT activity. On the other hand, the restored cells display a similar rod shape and PG profile that is indistinguishable from the uninduced control. Quantitative proteomics analysis of the restored cells identified significant protein enrichment in the regulator of capsule synthesis (Rcs) regulon, a two-component stress response known to be specifically activated by PG damage. We showed that LT-overexpressing *E. coli* with an inactivated Rcs system partially impairs the growth restoration process, supporting the involvement of the Rcs system in countering aberrant PG cleavage. Furthermore, we demonstrated that the elevated LT activity specifically potentiates β-lactam antibiotics against *E. coli* with a defective Rcs regulon, suggesting the dual effects of augmented PG cleavage and blocked PG synthesis as a potential antimicrobial strategy.

In all bacteria, the cell wall encases the cytoplasmic membrane and is essential for cell survival. The major constituent of the bacterial cell wall, peptidoglycan (PG), is made of long glycan polymers of alternating GlcNAc and *N*-acetylmuramic acid (MurNAc) residues, with a short stem peptide attached to each MurNAc unit (1). Peptides on adjacent glycan strands are cross-linked to rigidify the PG meshwork. PG serves as a protective exoskeleton that maintains the bacterial cell integrity against high internal osmotic pressure (2). However, PG is not a static structure and undergoes continuous enzymatic degradation and turnover during bacterial growth and division, allowing the insertion of new PG strands into the existing sacculus for cell expansion (3). The activity of such PG-degrading enzymes needs to be highly regulated to avoid uncontrolled lysis of the bacterial cells.

Lytic transglycosylases (LTs) are key cleavage enzymes that participate in bacterial PG turnover in Gram-negative bacteria (4). Belonging to the glycoside hydrolase family, LTs, however, specifically catalyze the nonhydrolytic cleavage of the glycosidic bond between MurNAc and GlcNAc in PG, generating 1,6-anhydromuramyl disaccharide muropeptides (5). In Gram-negative bacteria, soluble anhydro-muropeptides generated from LT activity are recycled back to the cytosol *via* the transporter AmpG and reused for PG synthesis, and these anhydro-muropeptides are also known effector molecules that induce β-lactamase expression *via* the AmpR-AmpC transcription system (6, 7, 8). *Escherichia coli* encodes eight LTs, which include six outer membrane–bound LTs (*i.e.* MltA, MltB, MltC, MltD, MltE, and MltF) and one soluble LT Slt70, and all seven are known to participate in PG degradation (9). The last LT, inner membrane–bound MltG, likely serves as a potential terminase to release nascent PG strands (10, 11). Biochemical characterization of individual LTs in *E. coli* has revealed significant redundancy among their activities *in vitro* (9). While genetic deletion of all *E. coli* LT genes is lethal, single or multiple KOs, including the sextuple deletion mutant, are all perfectly viable with normal growth rates and minimal changes of the PG structure, indicating the overlapping functions among the array of LTs in *E. coli* (4, 12).

The biological activity of bacterial LTs resembles that of the PG-degrading antimicrobial enzymes in nature, such as mammalian lysozymes. Lysozyme, which is abundantly present in mammalian tears, saliva, and mucous as an important innate immune defense mechanism, is a glycosidase hydrolase that hydrolytically cleaves the glycosidic bond between MurNAc and GlcNAc in bacterial PG, thus killing bacteria (13, 14, 15). To defend cell envelope integrity, bacteria have evolved several two-component systems that can sense and respond to PG damages and defects (16). Notably, Gram-negative bacteria such as *E. coli* possess the Rcs phosphorelay system (17, 18), which upregulates expressions of proteinaceous inhibitors of lysozyme, Ivy (inhibitor of vertebrate lysozyme) and MliC (membrane-bound lysozyme inhibitor of C-type lysozyme) as part of the stress response to overcome mammalian lysozymes’ insult on PG (19, 20, 21). Interestingly, these lysozyme inhibitors also suppress the glycosidase activity of *Pseudomonas aeruginosa* LT MltB *in vitro*, leading to the proposal that lysozyme inhibitors could be potential LT regulators in bacteria (22). However, the physiological relationship between lysozyme inhibitors in the Rcs pathway and bacterial LTs has not been directly demonstrated.

To uncover the potential regulatory mechanisms of bacterial LTs, we increased the periplasmic expression of MltA, MltB, or Slt70 individually in *E. coli*, which leads to cell rounding up due to the weakening of the PG. Unexpectedly, all LT-overexpressing *E. coli* cells can resume growth with normal rod morphology over time despite elevated LT levels. PG analysis revealed that the restored cells exhibit a similar PG profile compared to the uninduced controls, although induced *E. coli* releases a significantly higher amount of anhydro-muropeptides into culture supernatants, supporting the elevated LT activity. Quantitative proteomics analysis of the restored rod cells indicated significant upregulation of proteins under the Rcs regulon, including lysozyme inhibitors Ivy and MliC, presumably resulting from PG damage conferred by aberrant PG cleavage. We further showed that the inactivation of the Rcs pathway partially delayed the restoration process of the LT-overexpressing cells and demonstrated that both *E. coli* Ivy and MliC are weak LT inhibitors *in vitro* and unable to resist LT overexpression *in vivo*. Lastly, we established that augmented PG degradation by elevated LT activity potentiates the effects of β-lactam antibiotics in *E. coli* with a defective Rcs system, which highlights the antimicrobial potential of dual strategies to perturb bacterial PG metabolisms.

## Results

### Increased LT activity in *E. coli* triggers cell lysis followed by robust growth restoration

**Figure 1:**
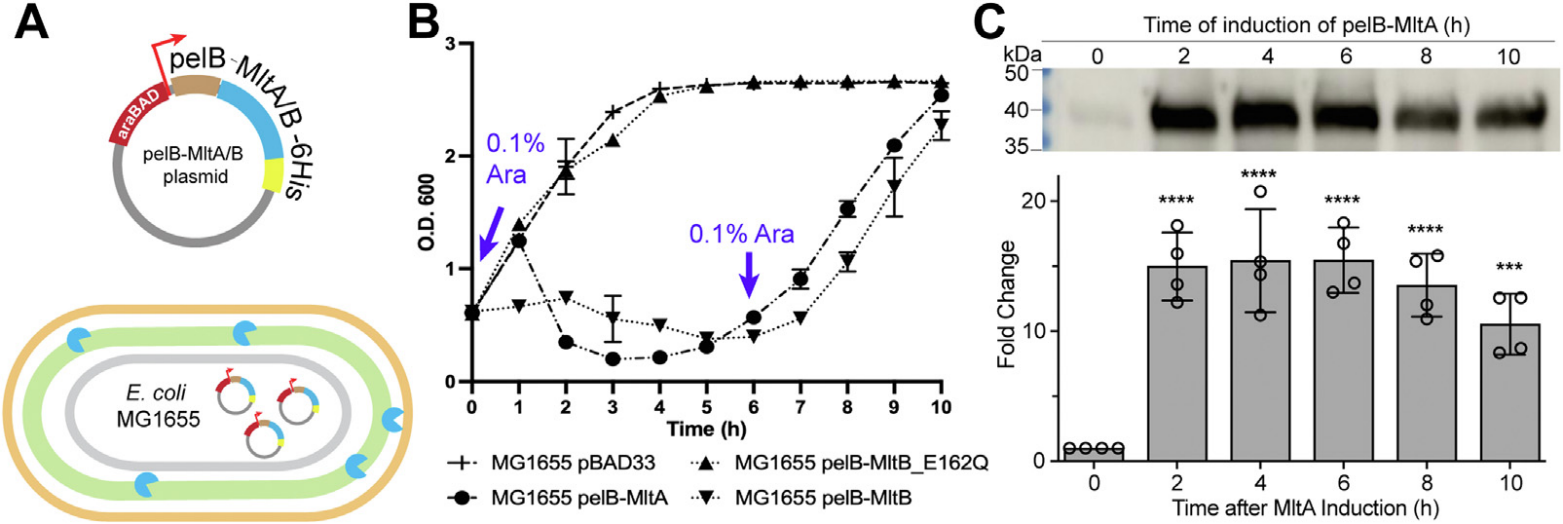
**Periplasmic overexpression of LT affects cell growth trends.** *A*, schematic of plasmid, pelB-MltA/B that is controlled under an araBAD promotor in *Escherichia coli*. *B*, growth curves. For *E. coli* harboring either pelB-MltA/B plasmids, the O.D. decreases upon L-Ara induction for 4 to 5 h, followed by robust cell growth restoration despite the addition of a second dose of L-Ara inducer. Overexpression of pelB-MltB_E162Q, the inactive mutant, does not affect growth. *C*, anti-His immunoblotting validates exogenous MltA expression in samples at different time points shown in *B*). The fold change of MltA expression was analyzed with ImageJ. Statistical analysis of the blots from at least three independent biological replicates was performed using ordinary one-way ANOVA. Results are presented as mean ± SD. ∗∗∗*p* <0.001, ∗∗∗∗*p* <0.0001. LT, lytic transglycosylase.

Due to the large number of redundant LTs in *E. coli*, it has been difficult to elucidate their cellular regulatory mechanisms. Genetic deletions of individual LT or multiple LTs in *E. coli* did not show obvious phenotypes (12). Herein, we decided to manipulate the system by augmenting the enzymatic activity of LT. We selected MltA and MltB, both of which are known to exhibit robust *in vitro* LT activity (9, 23), to be individually overexpressed in the periplasm of *E. coli*. For controlled expression, the respective *mltA* or *mltB* gene lacking the *N*-terminal membrane-anchoring region was cloned behind a pelB signal peptide to ensure periplasmic translocation and appended with a *C*-terminal His_6_ tag in the pBAD33 vector, yielding the pelB-MltA or pelB-MltB plasmid (Fig. 1*A*). While introducing pelB-MltA into *E. coli* MG1655 had no impact on cell growth, the addition of 0.1% l-arabinose (l-Ara) induced exogenous periplasmic MltA, effectively reducing culture optical density (O.D.) from 0.6 to 0.2 over several hours (Fig. 1*B*). A similar growth trend was observed upon pelB-MltB induction (Fig. 1*B*). We reasoned that the decrease in cell density is likely due to the degradation of PG exoskeleton by the increased LT activity, thereby weakening the protective layer and thus increasing lysis. On the other hand, periplasmic expression of the MltB active site mutant (pelB-MltB_E162Q) (24) did not alter cell growth (Fig. 1*B*). Put together, these observations indicate that the increased periplasmic LT activity, instead of LT expression level *per se*, impairs *E. coli* growth.

Intriguingly, upon withstanding the reduced cell density for 3 to 4 h, both the pelB-MltA– and pelB-MltB–expressing *E. coli* cultures were always able to resume growth, despite the sustained expression of the respective LT (Figs. 1*C* and S1, *C* and *D*). Moreover, taking the pelB-MltB–expressing *E. coli* as an example, we showed that the addition of different amounts of l-Ara inducers (0.02%, 0.1%, and 0.5%) all triggered O.D. reduction that was followed by robust growth restoration, with the rate of recovery inversely correlated to the amount of inducer used (Fig. S1*A*). Surprisingly, adding a second dose of l-Ara to the restored culture at O.D. ∼0.6 did not result in O.D. reduction again, suggesting that growth restoration was not due to insufficiency or depletion of l-Ara (Fig. 1*B*). Importantly, the sustained LT expression in these cells was confirmed by anti-His Western blotting (Figs. 1*C* and S1, *C* and *D*). These observations suggest that the restored *E. coli* culture may have developed robust intrinsic mechanisms to overcome the aberrant LT activity.

Notably, our observations of the robust growth restoration in LT-overexpressing *E. coli* culture differ from an earlier study by Ehlert *et al.* in 1995 (25), in which MltB overexpression led to rapid cell death within 10 min due to increased PG cleavage. In the previous work, native MltB was cloned behind the tac promoter in a pBR322-derived vector and induced with IPTG at an early log phase of O.D. ∼0.2 (25). We reasoned that culture growth disparity is likely due to different promoter strengths for LT expression (26, 27). In our system, the araBAD promoter was used and activated by intermediate concentrations of l-Ara inducer (0.02% to 0.5%), and thus, LT expression was likely to be milder, which may allow *E. coli* culture to restore growth over time instead of rapid lysis. Correspondingly, we observed significant culture clearance and cell debris when a high concentration of l-Ara (2%) was added. When MG1655-pelB-MltB was induced with 0.1 % l-Ara in the early log phase (O.D. ∼0.2), robust growth restoration was still observed (Fig. S1*B*), indicating that the effects are not caused by different induction time points. To rule out the growth trend as a potential artifact of altering MltA and MltB that are outer membrane–anchored LTs into periplasmic soluble proteins in our constructs, we evaluated the effects of overexpressing Slt70, the soluble periplasmic LT in *E. coli*. Indeed, MG1655-pelB-Slt70 cells manifest a similar growth curve of initial cell lysis followed by robust recovery upon induction (Fig. S1*E*). The fact that overexpression of either MltA, MltB, or Slt70, which belong to different LT families (4), gave rise to similar growth trends suggests that growth restoration is not specific to a particular LT but is likely a general response in *E. coli* to combat aberrant PG cleavage.

### Elevated LT activity alters *E. coli* cell morphology

**Figure 2:**
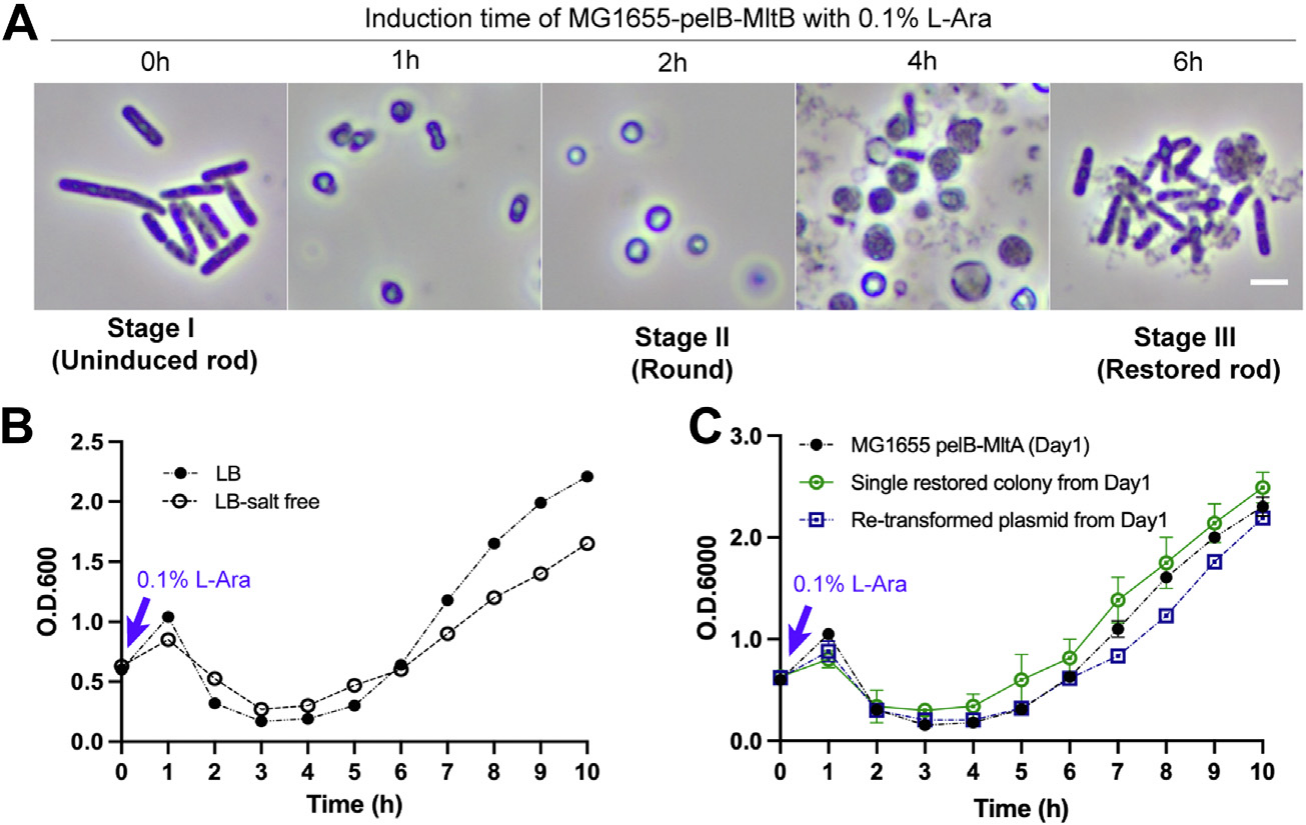
**Morphological changes of LT-overexpressing *Escherichia coli*.***A*, microscopy images of MG1655, harboring pelB-MltB taken at the indicated time points upon adding 0.1% L-Ara inducer. The uninduced rod *E. coli* cells (stage I) undergo rounding up (stage II) and lysis, followed by robust growth restoration with rod cells indistinguishable from the WT (stage III). Scale bar represents 1 μm. *B*, robust growth restoration of LT-overexpressing *E. coli* is observed under the hypotonic condition in LB-salt–free media. *C*, growth recovery of LT-overexpressing *E. coli* is not due to genetic suppressor mutations. Reinoculated single colonies from the restored culture and transformation of the isolated plasmids into MG1655 both give rise to characteristic growth trends, implying that genetic suppressors are not involved in restoration. LT, lytic transglycosylase.

The significant initial O.D. reduction of the pelB-MltA/B–expressing *E. coli* culture upon induction suggests cell lysis. Interestingly, in the initial hour after L-Ara addition, we observed that *E. coli* cells begin to round up with little cell debris, suggesting not much cell lysis at this stage. By 2 to 3 h, most cells have become spherical and continued to expand in size due to the high internal turgor pressure against the weakened cell wall (Fig. 2*A*). At this stage, we observed the most significant amount of cell debris, which is consistent with the lowest O.D. point observed in the growth curve. Colony forming units counting of viable cells also confirmed cell lysis at this stage (Fig. S2*A*). In addition, we also observed a small fraction of cells with a rod shape (<10%) among the lysed cell debris. After this period of cell lysis, both pelB-MltA/B–expressing *E. coli* cultures displayed restored growth with rod-shaped cells indistinguishable from the WT (Fig. 2*A*).

The morphological change in the LT-overexpressing *E. coli* is reminiscent of the formation and recovery of lysozyme-induced (LI) spheroplasts in previous reports (28). Ranjit and Young created spheroplasts by treating *E. coli* cells with lysozyme while under osmotic shock, effectively degrading the PG layer (28). LI spheroplasts were spherical in shape and 1.5× larger than normal rod cells. Remarkably, under isosmotic sucrose recovery conditions, most LI spheroplasts could revert to WT rod dimensions after 4 to 6 divisions (28). Thus, we asked if the growth restoration in our case could also be due to the recovery of spherical LT-overexpressing cells. However, time-lapse monitoring of the induced culture did not capture the cell reversion process but revealed that lysis was a common fate for spherical *E. coli* cells. We then reasoned that if the growth restoration indeed involves the recovery of the LT-overexpressing spherical cells, culturing them in hypotonic salt-free LB should promote lysis of the osmotically fragile spheroplasts and effectively prevent restoration (29, 30). To our surprise, both pelB-MltA/B–expressing *E. coli* strains still showed robust growth restoration when cultured in an LB-salt–free medium (Figs. 2*B* and S2*A*). Therefore, despite similar cell morphological changes, the growth restoration of LT-overexpressing *E. coli* culture is a distinct process from the LI-spheroplast recovery.

### Growth restoration of LT-overexpressing *E. coli* is not due to genetic suppression

We next explored if suppressor mutations could contribute to growth restoration. First, we obtained single colonies from the restored culture and reinoculated them individually into fresh LB to check for growth. Interestingly, upon LT induction, these cultures continued to show characteristic growth trends of lysis followed by restoration and displayed round and rod morphologies at the two stages, respectively (Fig. 2*C*). This implies that the restored rod cells do not possess genetic suppressor mutations (31), which would otherwise confer stable resistance against cell lysis induced by LT overexpression. Furthermore, to ensure the pelB-MltA/B plasmid is still intact and functional, we isolated plasmids from single colonies of the restored culture and introduced them back to MG1655 WT cells again. As expected, similar cell growth phenotypes and morphology were obtained with the retransformed plasmids (Fig. 2*C*). Correspondingly, sequencing of these plasmids isolated from the restored rod cells confirmed no mutational changes. Taken together, these observations rule out the possibility of suppressor mutations in the restored *E. coli* cells and in the LT-overexpressing plasmids (*i.e.*, pelB-MltA/B) that could potentially rescue cell lysis.

### Restored rod cells exhibit a similar PG profile as uninduced cells

**Figure 3:**
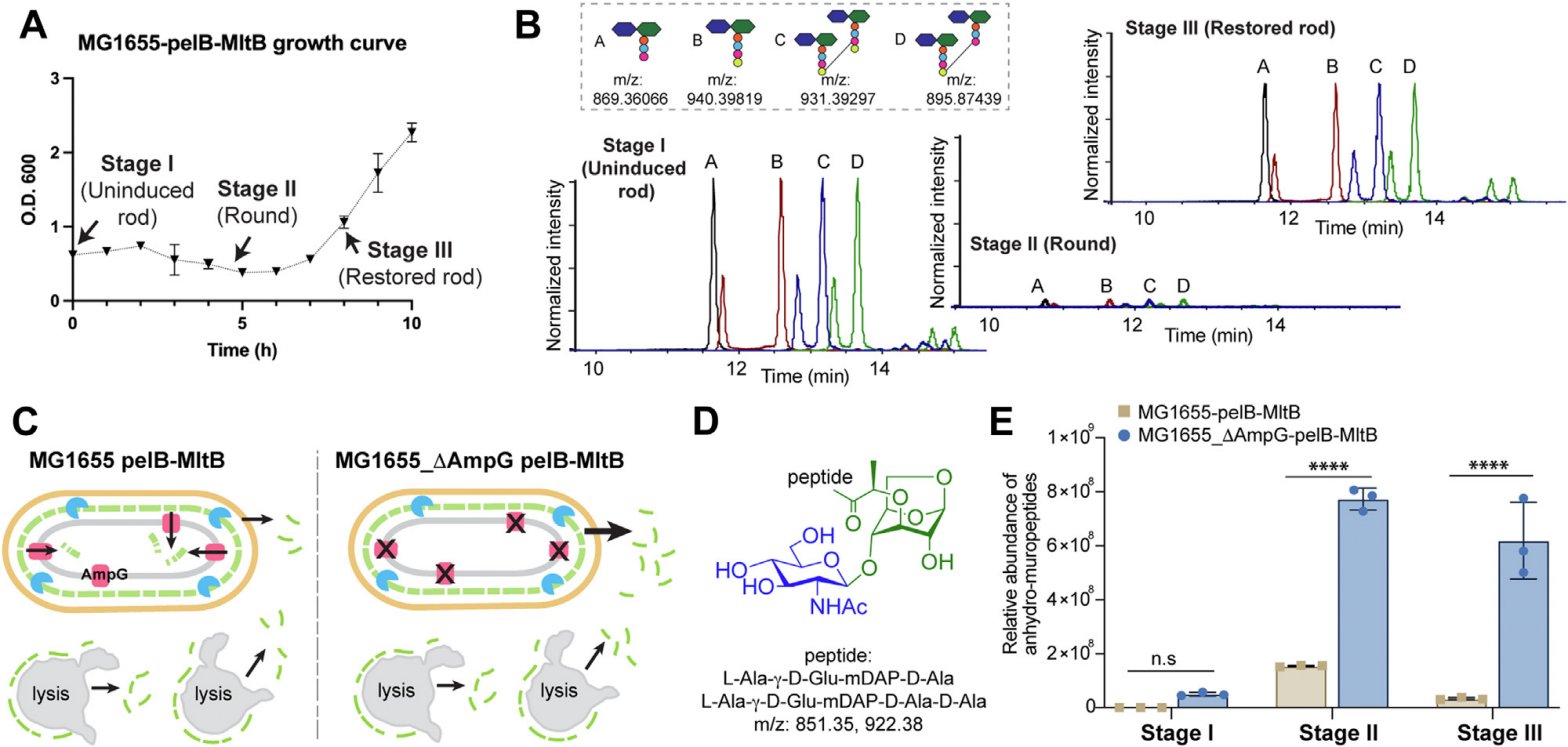
**Muropeptide analysis of LT-overexpressing *Escherichia coli* at three stages: uninduced rod (stage I), induced round (stage II), and restored rod (stage III).***A*, pelB-MltB–expressing *E. coli* MG1655 cells were harvested at different stages as indicated in the growth curve. The sample volume x O.D._600_ of each collection was normalized. *B*, LC-MS analysis of the PG profile of *E. coli* at the three stages. Stage II cells exhibit significantly lowered total ion abundance of muropeptides, supporting a weakened PG in these *spherical* cells; similar muropeptide compositions are found among all three stages. *C*–*E*, quantification of soluble anhydro-muropeptides in culture supernatants of MG1655 and MG1655_Δ*ampG* strains harboring pelB-MltB at the three stages. MG1655_Δ*ampG*, in which the cytoplasmic membrane anhydro-muropeptide transporter AmpG is deleted, releases an increased level of soluble anhydro-muropeptides into the media due to the blockage of the PG recycling pathway. This observation confirms the elevated periplasmic LT activity in cells upon pelB-MltB induction. Statistical analysis of three independent biological replicates was performed using ordinary one-way ANOVA. Results are presented as mean ± SD. ∗∗∗∗ *p* < 0.0001. LT, lytic transglycosylase; PG, peptidoglycan.

Intrigued, we next focused on elucidating any differences between the restored rod cells and the uninduced rod cells, which may offer potential clues on the cellular regulatory mechanisms that keep aberrant LT activity in check. Our first hypothesis was that the elevated LT activity might alter the PG profile in these bacteria (32, 33). To evaluate this, we harvested the pelB-MltB–expressing *E. coli* culture at three stages: uninduced rod, induced round, and restored rod (stages I-III, respectively) (Fig. 3*A*). The cell wall sacculi from each sample were digested with lysozyme, and the resulting crude muropeptide mixture was subjected to LC-MS analysis. Surprisingly, we did not observe any notable change in the overall PG composition (*i.e.*, average chain lengths and crosslinking levels) among the three stages, except for a significantly lower total ion abundance of muropeptides in the induced round cells, which is consistent with the weakened PG layer in these cells (Figs. 3*B* and S2, *B*–*E*). Next, we also showed that the expression level of the endogenous LTs in the restored cells was similar to that in the uninduced cells (Fig. S2*F*). Overall, our results show that although LT overexpression weakens bacterial PG and thus alters cell morphology, the restored rod cells do not exhibit notable alterations of the PG polymer compared to the uninduced cells. Similarly, a normal muropeptide composition was previously observed in *E. coli* L-form revertant cells too (34, 35).

### Release of soluble anhydro-muropeptides due to elevated LT activity

The elevated LT activity of the MltA/B-overexpressing *E. coli* should result in nonhydrolytic cleavage of the polymeric PG to generate soluble anhydro-muropeptide products (9, 30). Hence, we next explored if anhydro-muropeptides could be detected in the culture supernatants for all three stages (Fig. 3*A*). As expected, only the known LT products (*i.e.*, anhydro-muropeptides GlcNAc-ahMurNAc-l-Ala-γ-d-Glu-*m*DAP-d-Ala-d-Ala and GlcNAc-ahMurNAc-l-Ala-γ-d-Glu-*m*DAP-d-Ala; *m/z*: 922.38 and 851.35) (9, 23), were detected in the culture supernatants of both round and restored rod stages (stage II and III) of MG1655 pelB-MltB–expressing cells, whereas no detectable amount was found in the supernatant of uninduced rod cells (stage I) (Fig. 3, *C*–*E*). While the lysis of round cells possibly contributes to a substantial amount of anhydro-muropeptides in the culture medium, we wanted to determine if the anhydro-muropeptides were of periplasmic origin and directly generated from LT cleavage. Since *E. coli* is known to efficiently recycle its periplasmic anhydro-muropeptides back into the cytosol (6, 36), we resorted to the MG1655_Δ*ampG* strain, in which the cytoplasmic membrane transporter for anhydro-muropeptides, AmpG, is deleted, therefore releasing more periplasmic anhydro-muropeptides into the milieu (Fig. 3*C*) (36). When we introduced and induced the pelB-MltB plasmid in the MG1655_Δ*ampG* strain, we observed a growth trend similar to the WT (Fig. S2*G*). Indeed, we detected significantly higher levels of secreted anhydro-muropeptides in cultures from round and restored stages than those in its uninduced controls (Fig. 3, *C*–*E*). Given a similar level of cell lysis was observed in both pelB-MltB–expressing MG1655 and MG1655_Δ*ampG* strains upon induction, we attributed the higher abundance of anhydro-muropeptides present in the MG1655_Δ*ampG* culture supernatant in both round and restored rod cells (stage II and III) to the release of periplasmic anhydro-muropeptides. Taken together, our evidence implies that LT activity is elevated in the pelB-MltB–expressing strains upon induction.

### The LT-overexpressing *E. coli* shows upregulation of the Rcs stress pathway

**Figure 4:**
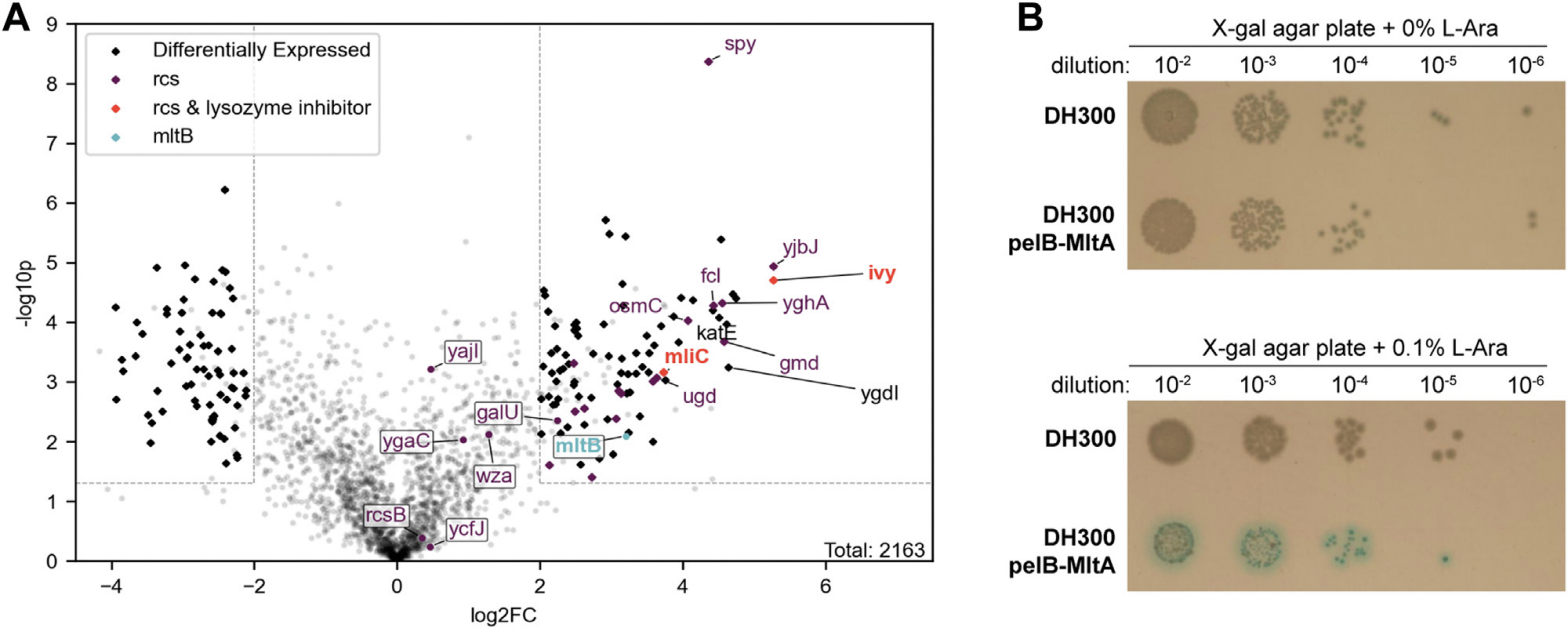
**Quantitative proteomics reveals the Rcs upregulation in the restored pelB-MltB–expressing MG1655 sample *versus* the noninduced control.***A*, volcano plot showcases the differentially expressed proteins, where proteins under Rcs regulon (highlighted in *purple*) are among the top upregulated hits. MltB is increased by 9-fold, confirming its overexpression in the pelB-MltB system. *B*, Rcs reporter strain DH300 (*rprA*-lacZ), harboring empty or pelB-MltA plasmid are plated on X-gal agar with or without L-Ara inducer. The increased *blue* color in DH300, harboring pelB-MltA plasmid on X-gal + 0.1% L-Ara plates confirms Rcs upregulation. Rcs, regulator of capsule synthesis.

To shed light on the potential coping mechanisms utilized by *E. coli* in response to LT overexpression, we performed iTRAQ-based quantitative proteomics analysis of the total cell lysates derived from both restored and uninduced *E. coli* cultures (Fig. S3*A*). As shown in the volcano plot in Figure 4*A*, subsets of proteins in the restored culture show significant differential expressions. Particularly, many upregulated proteins (shown in purple) belong to the Rcs regulon, including Ivy (∼38-fold), the uncharacterized lipoproteins YjbJ (∼38 fold), periplasmic chaperone Spy (∼20 fold), and MliC (∼13-fold). The Rcs regulon encodes a two-component system that responds to cell envelope defects and is specifically activated during PG stress conditions (16, 17, 18), such as exposure to lysozyme and β-lactam antibiotics (21, 37, 38). Thus, it is reasonable that the Rcs pathway is upregulated as a stress response to the elevated LT activity in pelB-MltA/B–expressing *E. coli*. Importantly, we noted that MltB (shown in blue) was moderately increased (∼9-fold), validating its overexpression in the pelB-MltB system.

We reasoned that if Rcs upregulation pertains to the elevation of LT activity in *E. coli*, they should be solely activated in the pelB-MltB–expressing strain but not the pelB-MltB_E162Q mutant (Fig. S3*A*). As expected, none of the Rcs hits was identified in the strain harboring the catalytically inactive pelB-MltB_E162Q plasmid, implying that the protein enrichment was not a result of higher MltB expression level *per se* but a response to the elevated LT activity. Comparing protein levels in the restored pelB-MltB–expressing cells *versus* the pelB-MltB_E162Q mutant also yielded a similar set of upregulated proteins of the Rcs regulon (Fig. S3, *B*–*D*). Overall, proteomic analysis established significant enrichment of the Rcs regulon in the restored *E. coli* cells.

To experimentally validate Rcs upregulation in cells with elevated LT activity, we introduced the pelB-MltA/B or pelB-MltB_E162Q plasmid into *E. coli* DH300 reporter strain (39), which harbors a *lac*Z reporter gene fused with *rprA*. RprA is a small RNA regulator encoded under the Rcs regulon (39), and this reporter strain has been widely used to study Rcs activation (21, 38, 39, 40). Upon plating the pelB-MltA/B–expressing reporter strain on X-gal plates containing 0.1% L-Ara, we indeed observed β-galactosidase activity indicating significant Rcs induction (Figs. 4*B* and S3*F*). Besides the Rcs regulon, gene ontology analysis of the differentially expressed proteins also revealed changes in several other pathways. For instance, proteins involved in aerobic respiration, production of colanic acid and extracellular polysaccharide precursors, and oxidative/hyperosmotic stress response were upregulated, while proteins involved in ribosomal and organelle assembly were downregulated (Fig. S3*E*). Hence, the proteomics profile of the restored rod cells is consistent with a population undergoing cell envelope damage that further leads to oxidative and osmotic stresses (41, 42, 43). Moreover, we confirmed that the restored cells exhibit a slightly higher, albeit insignificant, amount of colanic acid, a component of the negatively charged polysaccharide capsule also known to be regulated under the Rcs system in *E. coli* (Fig. S3*G*) (44). Taken together, we experimentally validated Rcs upregulation in the restored culture of the LT-overexpressing *E. coli*, confirming the proteomics results.

### Lysozyme inhibitors, Ivy and MliC in the Rcs regulon partially inhibit *E. coli* LT activity *in vitro*

**Figure 5:**
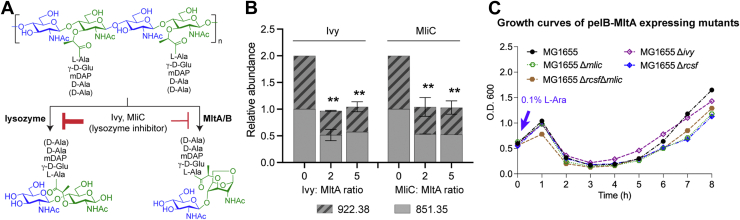
**Lysozyme inhibitors Ivy and MliC in the Rcs regulon partly contribute to resistance against elevated LT activity in *Escherichia coli*.***A*, lysozyme and LTs act on the same PG substrate. Proteinaceous lysozyme inhibitors Ivy and MliC are weak inhibitors against *E. coli* MltA and MltB *in vitro*. *B*, LC-MS quantification of anhydro-muropeptide products generated by recombinant MltA in the absence or presence of an excess amount of Ivy or MliC. Both lysozyme inhibitors only exert partial inhibitory effects despite the stoichiometric excess. *C*, growth trend of pelB-MltA–expressing *E. coli* MG1655 and mutants lacking *ivy*, *mlic*, *rcsf*, or both of the latter. Upon induction of 0.1% L-Ara, all mutants show characteristic growth trends and morphological changes as the WT, while the Δ*rcsf* mutant consistently shows slightly delayed restoration. Results are presented as mean ± SD. ∗∗ *p* < 0.01. PG, peptidoglycan; LT, lytic transglycosylase; Rcs, regulator of capsule synthesis; MliC, membrane-bound lysozyme inhibitor of C-type lysozyme; IVY, inhibitor of vertebrate lysozyme.

Among the top enriched protein candidates in proteomics results, our attention was drawn to two lysozyme inhibitors, Ivy and MliC, in the Rcs regulon (Fig. 4*A*). Both are potent proteinaceous inhibitors known to act against C-type lysozymes, such as hen egg white lysozyme, as part of the putative virulent factors in Gram-negative bacteria to fend off vertebrate innate immunity (19, 20, 21). Since bacterial LTs act on the same PG substrate as vertebrate lysozymes, we wondered if these proteinaceous lysozyme inhibitors could also inhibit LT activity (Fig. 5*A*). Interestingly, Clarke et al. had previously demonstrated that *P. aeruginosa* lysozyme inhibitors, Ivyp1 and Ivyp2 could inhibit *P. aeruginosa* MltB *in vitro* (22). Herein, the significant enrichment of both Ivy and MliC in the restored LT-overexpressing *E. coli* culture led us to hypothesize that *E. coli* might utilize these lysozyme inhibitors as an intrinsic resistant mechanism to keep the elevated LT activity in check.

To test this hypothesis, we next set up an LC-MS assay to evaluate the *in vitro* PG glycosidase activity of recombinant MltA with isolated PG sacculi by monitoring the quantity of signature anhydro-muropeptide products (A: m/z = 922.38; B: m/z = 851.35) at different incubation time intervals. As expected, *E. coli* MltA exhibits excellent enzymatic kinetics *in vitro* (9), since the formation of anhydro-muropeptide products reaches saturation within 20 min (Fig. S4*A*). To evaluate the inhibitory effect of each lysozyme inhibitor, we incubated MltA with either Ivy or MliC at varying stoichiometric ratios and quantified the amount of anhydro-muropeptides at the end time-point. As shown in Figure 5*B*, while Ivy and MliC partially inhibited MltA activity *in vitro*, both did not exhibit any inhibitory effects when heat-quenched (Fig. S4*B*). Combining Ivy and MliC did not improve the inhibitory effects, and similar partial inhibitions were observed for the recombinant MltB as well. Hence, the specific yet incomplete inhibition of both Ivy and MliC against *E. coli* MltA/B suggests that they are only weak LT inhibitors and unlikely the major resistant mechanisms against elevated cellular LT activity. Consistent with their weak binding affinity (if any), we could not detect any direct protein–protein interactions between both lysozyme inhibitors and MltA/B protein *in vitro*.

To evaluate the physiological relevance of lysozyme inhibitors of the Rcs regulon in overcoming elevated LT activity in *E. coli*, we introduced the pelB-MltA plasmid into MG1655 single KO mutants of *ivy*, *mliC*, and *rcsf*, respectively. RcsF is the upstream sensor of the Rcs pathway whose deletion should inactivate the entire regulon (17, 18, 45, 46, 47). Upon induction, all these mutants harboring pelB-MltA exhibit characteristic growth trends of lysis followed by growth restoration, similar to the MG1655 WT (Figs. 5*C* and S4, *C*–*E*). Notably, we observed that the Δ*rcsf* mutant consistently showed slightly delayed restoration in both liquid culture and on agar plate (Fig. S4*F*), supporting the potential involvement of Rcs in the growth restoration process. Nevertheless, the fact that all mutants could eventually restore growth implies that Rcs is not the sole intrinsic resistant mechanism against aberrant LTs. On the other hand, overexpression of lysozyme inhibitors (Ivy and MliC) in MG1655 is also insufficient to confer resistance against cell lysis induced by the aberrant LT activity (Fig. S4*G*). Thus, our observations are in line with the existence of other, hitherto unidentified, LT-inhibitory factors in *E. coli*.

### Elevated LT activity sensitizes β-lactams against *E. coli* with an inactivated Rcs regulon

**Figure 6:**
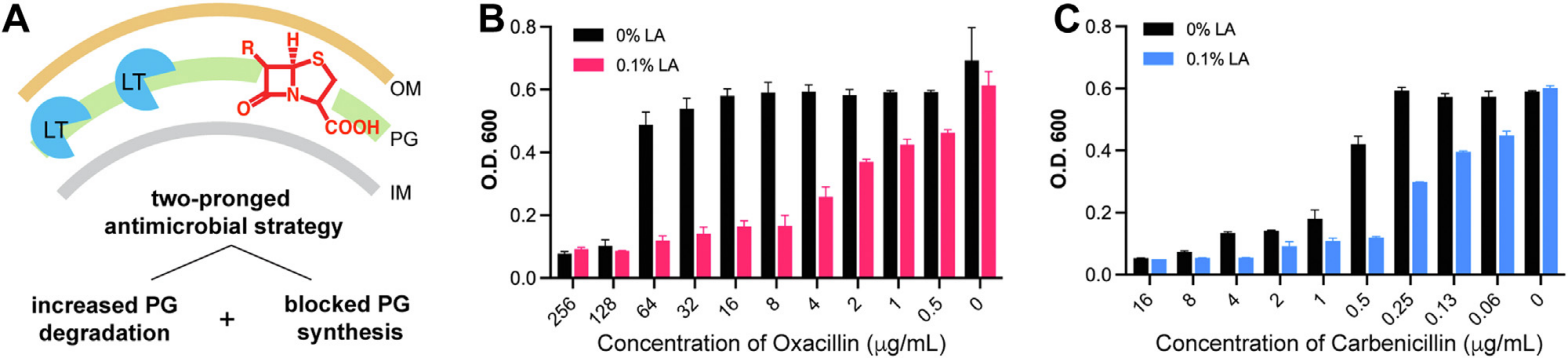
**Combination of LT elevation and β-lactam treatment confer sensitivity in *Escherichia coli*.***A*, schematic of the two-pronged strategy to enhance PG degradation by LT and inhibit PG synthesis by β-lactam drugs in *E. coli*. *B* and *C*, induction of pelB-MltA in MG1655_Δ*rcsf* significantly sensitizes it to β-lactam drugs, oxacillin and carbenicillin, by ∼16-fold. PG, peptidoglycan; LT, lytic transglycosylase.

The Rcs stress response protects bacteria against deleterious effects on PG (18). Previous studies have revealed that β-lactam inhibition or genetic inactivation of different penicillin-binding proteins (PBPs) trigger Rcs response in *E. coli* (37, 38, 48). Here, we have shown that the LT-overexpressing *E. coli* also manifests Rcs upregulation. Thus, we wondered if LT elevation to augment aberrant PG degradation combined with inhibition of PG synthesis by β-lactams together may confer potent antimicrobial effects (Fig. 6*A*). Indeed, MG1655_Δ*rcsf* was drastically sensitized towards β-lactam antibiotics upon the induction of pelB-MltA. Specifically, the minimum inhibitory concentration (MIC) of oxacillin against MG1655_Δ*rcsf* decreased from 128 μg/ml to 8 μg/ml, manifesting a nearly 16-fold increase in sensitivity when LT activity is elevated (Fig. 6*B*). Similar sensitization effects were observed for other β-lactams, such as carbenicillin and penicillin G, while the elevated LT activity had no impact on kanamycin and colistin, which are non-PG–targeting antibiotics (Fig. S5). Different from the previous study which showed that Rcs inactivation led to cell plating defects on agar containing the specific β-lactam (38), in our system, MG1655_Δ*rcsf* did not display reduced cell survival under β-lactam treatment alone (compared to MG1655 WT), yet it manifested significant β-lactam sensitization upon LT elevation.

Thus, without Rcs protection, *E. coli* exhibits increased sensitivity towards the dual effects of LT hyperactivity and β-lactam treatment. The increase of LT activity to enhance aberrant PG degradation combined with the inhibition of PG synthesis could offer a potential two-pronged antimicrobial strategy in *E. coli*.

## Discussion

PG is essential against osmotic lysis. Bacteria encode a large number of enzymes in both PG assembly and disassembly processes, which need to function in a highly coordinated manner to prevent the disruption of PG structural integrity (3). On the one hand, cellular regulations of PG synthases have been relatively well characterized in rod-shape Gram-negative bacteria such as *E. coli*, in which the major PBPs are localized in either the elongasome or divisome multiprotein complexes for regulated PG synthesis during bacterial lateral growth or division, respectively (49). In addition, recent studies have revealed that the activities of PG synthases, PBP1a and PBP1b, are specifically stimulated by outer membrane lipoproteins, LpoA and LpoB (50, 51, 52, 53, 54), showcasing the existence of bacterial intrinsic regulatory machinery for PG synthesis. On the other hand, LTs are critical enzymes that degrade PG polymers and generate soluble PG fragments to accommodate bacterial growth and division (5, 30). Although aberrant LT activity can be lethal, their cellular regulatory mechanism is not clear, and this is partly complicated by the functional redundancy of multiple LTs in bacteria (4, 30). Earlier studies have demonstrated *in vitro* interactions between *E. coli* MltA and PBP1B that is mediated by a novel MltA-interacting protein, Mip (55), and between *P. aeruginosa* SltB1 and the N-terminal noncatalytic domain of PBP2 (56), as well as recent *in vivo* evidence of *E. coli* MltG–PBP1B interaction in the bacterial adenylate cyclase two-hybrid assay (10). However, the relevance of such protein–protein interactions to the control of LT activity has not been demonstrated *in vitro* or *in vivo.* Hence, how *E. coli* and other Gram-negative bacteria regulate the lethal activity of LTs for PG maintenance, and integrity has remained elusive. Decoding the regulatory mechanisms might provide new antimicrobial opportunities targeting LTs.

In this study, we showed that periplasmic overexpression of LT, MltA or MltB, in *E. coli* induces cell rounding and lysis due to the weakening of PG. While the native MltA and MltB in *E. coli* are both palmitoylated proteins anchored onto the outer membrane (25, 32, 33), we constructed pelB-MltA/B systems that would translocate MltA/B into the periplasmic space and release them as soluble LTs instead (57). Our initial trials to overexpress full-length MltA/B in the pBAD33 vector in MG1655 did not yield any phenotype, whereas the induction of pelB-MltA/B (cloned in pBAD33) in *E. coli* led to remarkable growth phenotypes and morphological changes. We attributed this observation to the following: (1) the pelB signal peptide is likely more efficient at periplasmic translocation of the target proteins where PG substrates are located and/or (2) the production of MltA/B as soluble LTs in the periplasmic space enhances their enzymatic activity since their localizations are no longer confined. In addition, we confirmed that the overexpression of the soluble periplasmic LT Slt70 of *E. coli* also affords similar growth effects. Moreover, we showed that periplasmic overexpression of the MltB active site mutant (MltB_E162Q) (24) in the pelB-MltB_E162Q construct does not affect cell growth at all, supporting that cell rounding up is a result of PG cleavage by the elevated LT activity, rather than the periplasmic build-up of overexpressed proteins. Thus, our pelB-MltA/B constructs enabled us to probe the phenotypes and effects of elevated LTs in *E. coli*.

While the MltA/B-overexpressing *E. coli* undergoes similar morphological changes (*i.e.*, rounding up then back to rod shapes) as LI spheroplasts, the culture restoration processes appear distinct. In a previous study by Ranjit and Young (28), *E. coli* LI spheroplasts reverted to rod shape after 4 to 6 generations in a process dependent on Rcs but independent of lysozyme inhibitors such as Ivy or MliC. In contrast, in our study, the growth restoration of LT-overexpressing *E. coli* readily took place in both LB and hypotonic LB-salt–free conditions, suggesting the growth restoration is not due to the reversion of spherical cells, unlike the previous study. Instead, restoration is likely a result of the continuous division of the residual MltA/B-overexpressing rod cells that did not round up initially. As gene expression from the araBAD-containing plasmid was known to be heterogenous (58), we suspect that the initial ‘resistant’ rod cells, representing a small number of the pelB-MltA/B–expressing *E. coli* population, express LT at a milder level that is sufficient to induce Rcs stress response but not enough to effectively alter cell morphology and induce lysis. These cells appear to be unaffected by induction and eventually take over the whole population while most of the LT-overexpressing cells are lysed. Immunoblotting shows that the restored rod cells still express exogenous LT significantly, albeit to a slightly lower level than the round cells, as expected. Additionally, the significant downregulation of ribosomal assembly processes in the restored cells (as elucidated by proteomics results), which is possibly a result of increased oxidative stress due to a breached cell envelope (59), may further restrain exogenous LT overexpression. Importantly, we showed that pelB-MltA/B plasmids in the restored rod cells remain functional, ruling out potential contributions of genetic suppression mutations.

Quantitative proteomics analysis of the restored culture revealed a significant enrichment of proteins in the Rcs regulon and downregulation of ribosomal assembly. The Rcs phosphorelay is a stress system that is specifically activated by PG damage (16, 17, 18). Previous studies have identified Rcs activation under PG stress conditions *via* the global gene expression analysis (38, 60). Herein, our proteomics findings also corroborate with Rcs upregulation in LT-overexpressing *E. coli*. However, the molecular details of how bacteria sense different kinds of cell envelope stresses and activate the Rcs regulon are currently unclear (46). Interactions between the outer membrane lipoprotein RcsF and the inner membrane sensor kinase RcsC are critical for signal transduction and downstream activation of the transcription regulators in the cytosol (17, 18). In our pelB-MltA/B–expressing *E. coli*, the elevated LT activity leads to aberrant PG degradation and hence the release of abundant soluble anhydro-muropeptides in the periplasmic space. Similarly, β-lactam antibiotics, also known to trigger Rcs response (38), induce a futile cycle of PG synthesis and degradation, during which LTs cleave the nascent PG strand to generate high levels of soluble anhydro-muropeptides in cells (61). Thus, we postulate that such an abnormally high amount of periplasmic muropeptides may serve as the molecular cues that RcsF detects. It would be interesting to determine if muropeptides alter binding interactions between RcsF and its negative regulator IgaA (40, 62), as well as the downstream histidine kinase RcsC for phosphorelay activation (46). Alternatively, perturbation of PG structures may also induce periplasmic osmotic pressure and/or changes in membrane integrity and curvature, which could, in turn, activate the Rcs regulon (18).

In the restored rod *E. coli* cells, we identified upregulated proteins such as Ivy, Yjbj, Ygdl, Spy, and MliC, which are components of the Rcs regulon (38). While the functions of several ‘y-genes’ in *E. coli* remain uncharacterized (63), the enrichment of Ivy and MliC, two putative lysozyme inhibitors (19, 20, 21), appears appealing. Such lysozyme inhibitors are only encoded by certain Gram-negative bacteria lacking the ability to *O*-acetylate the C-6 hydroxyl group on MurNAc residues in the PG backbone (64), a structural modification that renders resistance to lysozyme and autolytic LTs. The paradox that bacterial lysozyme inhibitors are localized in the periplasm in Gram-negative bacteria, a physically separated space from the external vertebrate lysozymes unless the bacterial outer membrane is breached, has led to the proposal that the physiological functions of lysozyme inhibitors are to regulate LT-induced autolysis instead (38, 65). Interestingly, Clarke et al. demonstrated that both *P. aeruginosa* Ivyp1 and Ivyp2 paralogs inhibit its MltB *in vitro* (22). However, evidence that supports their relationships *in vivo* has been lacking. In our current study with MltA/B-overexpressing *E. coli*, we found that both Ivy and MliC (among other Rcs proteins) were upregulated as a stress response in LT-overexpressing *E. coli*. Similarly, we showed that both Ivy and MliC could partially inhibit *E. coli* MltA/B activity *in vitro*, despite their incomplete inhibitory effects at high molar excess. We duly note that our LC-MS analysis differs from the assay setup by Clark et al., in which they measured O.D. decrease of sacculi digestion as a readout of LT activity (22), whereas our LC-MS analysis is inevitably more sensitive at detecting residual anhydro-muropeptide products of *in vitro* enzymatic reactions. Nevertheless, Clark et al. also reported that at least a 13-fold excess of the lysozyme inhibitors was needed to effectively inhibit *P. aeruginosa* MltB activity *in vitro* (22). Thus, our findings agree that these lysozyme inhibitors are functional but relatively weak against bacterial LTs. Consistently, *E. coli* mutants lacking either or both *ivy* and *mliC* can still restore growth upon the induction of pelB-MltA/B, while overexpression of the lysozyme inhibitors is not sufficient to confer resistance against cell lysis induced by aberrant LT activity, suggesting that they may not be the key intrinsic inhibitors against LTs *in vivo*. On the other hand, the inactivation of the entire Rcs system (*i.e.*, Δ*rcsf* mutant) causes slightly more pronounced delays in culture restoration, supporting that the Rcs system as a whole is partly involved in countering the aberrant LT activity.

Importantly, altering the PG cleavage activity of LTs in *E. coli* affects its β-lactam resistance. On the one hand, the recycled cytosolic anhydro-muropeptides from LT activity act as signaling molecules to activate the *ampC-ampR*–encoded β-lactamase production in Gram-negative bacteria (6). The *E. coli* mutant lacking major LTs including MltA and MltB, which reduces its cellular PG turnover, dramatically suppresses β-lactamase induction and is sensitized towards certain β-lactam antibiotics (66, 67). These studies implied that inhibition of LT activity in *E. coli* could synergize with β-lactam antibiotics in a β-lactamase–dependent manner (68). On the other hand, we have shown that the induction of exogenous LTs could also effectively potentiate β-lactam antibiotics in *E. coli* with a defective Rcs system. While both LT elevation and β-lactam treatment are known to trigger Rcs stress separately (38), *E. coli* MG1655_Δ*rcsf* strain with an inactivated Rcs regulon does not exhibit increased sensitivity towards either treatment alone. However, a combination of increased PG cleavage by elevated LT activity and inhibition of PG synthesis by β-lactams together effectively impedes the growth of the MG1655_Δ*rcsf* mutant. Herein, we demonstrate the lethal effects of augmented PG cleavage and blocked PG synthesis in the Rcs-inactivated *E. coli* background. While the mechanisms of such synergistic effects require further investigations, our observations are in line with the involvement of the Rcs regulon in countering PG damage, since *E. coli* without the Rcs system is greatly sensitized. Interestingly, Bernhardt et al. previously reported that the overexpression of *E. coli* LT MltG in a multicopy plasmid is synthetic lethal with the genetic inactivation of *pbp1b*, suggesting the intricate connection and balance between PG synthesis and cleavage necessary for bacterial survival (10, 69). Consistently, we also showed that the sensitization effect of elevated LT activity is specific to β-lactams but not other non-PG–targeting antibiotics such as kanamycin and colistin. Taken together, our work support that decoupling the fine balance between PG cleavage and PG synthesis in bacteria might lead to potential two-pronged antimicrobial strategies.

## Experimental procedures

### Bacterial strains, plasmids, and media

The bacterial strains and plasmids used in this study are listed in Tables S1 and S2, respectively. PCR was performed by KOD Hot Start DNA polymerase (National England BioLabs/NEB) or Q5 High-Fidelity 2× Master Mix (NEB) for cloning purposes according to the manufacturer’s instructions. Plasmid DNA and PCR fragments were purified using the plasmid miniprep kit (NEB) and DNA gel extraction kit (NEB). *E. coli* K12 chromosomal DNA was used as the template. *E. coli* DH5α stain was used for cloning, and *E. coli* BL21 (DE3) was used for recombinant protein purification. *E. coli* MG1655 strain and KO mutants were used for growth curve measurement, PG composition analysis, immunoblotting, and MIC study.

Unless otherwise specified, bacteria were cultured in LB [1% tryptone, 0.5% yeast extract, 0.5% NaCl] or LB-salt–free [1% tryptone, 0.5% yeast extract] medium at 37 °C. Agar 1.5% (w/v) was used in solid plates. Antibiotics were used at the following concentrations (per mL): 30 (chloramphenicol; Cm30), 50 (kanamycin; Kan50), or 100 (ampicillin; Amp100) where appropriate.

### Construction of LT-overexpressing strains

The pET22b_pelB-MltA/MltB-6H plasmids were constructed initially by cloning the mltA and mltB gene lacking the respective signal peptide into pET-22(b)+ vector *via* Gibson assembly. The pelB_MltA/B-6H fragments were then subcloned into a modified pBAD33 vector that contains a ribosome binding site, yielding pBAD33-RBS-pelB-MltA[24-365]-6H and pBAD33-RBS-pelB-MltB[22-361]-6H plasmid, respectively. The pBAD33_RBS_pelB-MltB-E162Q plasmid was generated *via* Q5 Site-Directed Mutagenesis Kit (NEB).

The pET22b_pelB-slt70[28-654]-6H plasmid was constructed *via* restriction enzyme cloning of the insert encoding slt70[28-654] to pET22b backbone at EcoRI and XhoI sites. The pelB-slt70[28-645] fragment was subcloned into the pBAD33-RBS vector, yielding pBAD33-RBS-pelB-slt70[28-645]-6H plasmid. The sequences of the resulting plasmids were confirmed (Biobasic sequencing) and introduced into *E. coli* MG1655 or the respect KO mutants *via* heat-shock transformation. The colonies are selected on LB Cm30 plates.

For overexpression of lysozyme inhibitors, the full-length *ivy/mliC* genes were amplified from *E. coli* K12 genomic DNA and cloned into pTrc99A vector between the EcoRI and HindIII sites, yielding pTrc99A_ivy/mlic-6H plasmids. The sequences of the plasmids were confirmed (Biobasic sequencing) and cotransformed with pBAD33-RBS-pelB-MltA[24-365]-6H into *E. coli* MG1655. The colonies are selected on LB Amp100+ Cm30 plates.

### Growth curves and morphological analysis

*E. coli* strains harboring pBAD33-RBS-pelB-MltA[24-365]-6H, pBAD33-RBS-pelB-MltB[22-361]-6H, or pBAD33-RBS-pelB-Slt70[28-645]-6H plasmids were routinely grown at 37 °C with aeration in LB medium supplemented with appropriate antibiotics. For growth curve studies, overnight saturated culture (250 μl) was diluted into LB supplemented with Cm30 (25 ml). The starter culture was incubated at 37 °C with shaking at 220 rpm until its O.D._600_ reached 0.60 ± 0.05, where a final concentration of 0.1% L-Ara (Sigma-Aldrich) was added. Subsequently, 1 ml of the culture was removed hourly for O.D._600_ measurement using a NanoDrop One Spectrophotometer (Thermo Fisher Scientific). Different induction O.D._600_ and L-Ara concentrations were tested as described in the figure legends (Fig. S1). For morphological analysis, 100 μl of the bacterial culture was fixed with 10 μl of 37 % formaldehyde (Alfa Aesar) and viewed using microscopy (Olympus CX43) at 100× magnification using the BASLER Microscopy Software (https://www.baslerweb.com/en/downloads/software-downloads/basler-microscopy-software/).

For growth curve measurements of the induction of MltA in Ivy/MliC-overexpressing *E. coli*, the overnight cultures of *E. coli* (harboring pTr99a-ivy-6H + pBAD33-RBS-pelB-MltA[24-365]-6H or pTr99a-mliC-6H + pBAD33-RBS-pelB-MltA[24-365]-6H or pTr99a-ivy-6H + pTr99a-mliC-6H + pBAD33-RBS-pelB-MltA[24-365]-6H) were diluted into LB supplemented with Cm30, Amp100, and IPTG (final 100 μM) to an O.D._600_ 0.1. The culture was incubated at 37 °C with shaking at 220 rpm until its O.D._600_ reached 0.60 ± 0.05, where a final concentration of 0.1% L-Ara (Sigma-Aldrich) was added for MltA induction. The O.D._600_ of the culture is monitored hourly.

### Immunoblotting

*E. coli* culture from the growth curve study was collected at various time points for analysis of LT overexpression by Western blot. Samples were normalized (O.D._600_ x Vol is same) and centrifuged (10,000*g*, 20 min) to collect cell pellet, which was then resuspended in 1× PBS (40 μl) and 5× Laemmli lysis buffer (10 μl) containing 5% β-mercaptoethanol. The sample was boiled at 100 °C for 5 min for lysis and loaded (20 μl) onto a 12% SDS-PAGE gel (with 4% stacking gel) for protein separation. The gel was transferred to a polyvinylidene difluoride membrane (Bio-Rad) and blocked with TBST buffer (0.1% Tween 20 in 1 × Tris-Buffered Saline, pH 7.6) with 3% nonfat skim milk for 1 h at room temperature with rocking. After several washes, the membrane was incubated with HRP Anti-6x His tag antibody (proteintech, 66005-1-Ig) at a 1:10,000 dilution in TBST buffer containing 5% bovine serum albumin for 1 h at room temperature. After several washes, the immunoblot was developed using Pierce ECL Western Blot substrate (Thermo Fisher Scientific) and imaged chemiluminescence using Amersham ImageQuant 800 (GE Healthcare). Similarly, for *E. coli* overexpressing both lysozyme inhibitors and MltA, the samples were collected and analyzed as above to validate the coexpression of Ivy/Mlic with MltA.

### LC-MS/MS analysis of PG profile

Bacterial sacculi were isolated based on previously described protocol (70) with some modifications. Briefly, the LT-overexpressing *E. coli* culture was collected (at least 200 ml) at different time points upon induction with 0.1% L-Ara. The exact volume collected was adjusted with O.D. of each sample, such that Vol × O.D. was consistent for all samples. The cell pellet was collected by centrifugation (5250*g*, 10 min) and resuspended in boiling SDS solution (0.5% v/v, final) for 30 min. The sacculi were collected by centrifugation (15,000*g*, 10 min) and washed repeatedly with deionised (D.I.) water for complete removal of residual SDS. The washed sacculi were then resuspended in trypsin (50 μg/ml in D.I. water, final) to incubate at 37 °C for 1 h with shaking. Upon heat inactivation of trypsin (100 °C, 10 min), the sacculi were washed and resuspended in 200 μl digestion buffer (12.5 mM NaH_2_PO_4_, pH 5.5) containing muramidase (100 μg/ml, final) for 16 to 18 h at 37 °C with shaking at 200 rpm. The enzymatic mixture was heat-inactivated (100 °C, 10 min), centrifuged (15,000*g*, 20 min), and supernatant was collected containing soluble muropeptides for LC-MS/MS analysis.

The LC-MS/MS analyses were performed using Vanquish 3000 HPLC coupled with Orbitrap Exploris 120 MS (Thermo Fisher Scientific), equipped with the C_18_ reversed phase Waters Symmetry Shield column (150 × 3 mm, 100 Å, 3.5 μm) with matching guard column. Muropeptides were eluted at a flow rate of 0.25 ml/min as follows: isocratic elution with 99% solvent A (HPLC/LC grade water with 0.05% formic acid) and 1% solvent B (HPLC/LC acetonitrile with 0.05% formic acid) for 3 min, a 20 min linear gradient to 40% B, a 1 min linear gradient to 90% B, a 5 min isocratic elution with 90% B, a 1 min linear gradient back to 1% B, and a 5 min isocratic elution with 1% B. The representative ion species derived from each muropeptide were extracted and integrated from total ion chromatograms using Xcalibur Qual Browser (Thermo Fisher Scientific, version 4.2) to compare the relative abundance of muropeptides among different samples.

### Analysis of soluble pool of anhydro-muropeptide in culture medium

LT-overexpressing *E. coli* cultures at different stages were grown to representative O.D._600_ and cooled on ice for 10 min. Samples were normalized (O.D._600_ x Vol is the same) and harvested by centrifugation (5000*g*, 10 min, 4 °C). The supernatant collected (6 ml) was treated with 3% TFA for protein precipitation and removal. Upon centrifugation (5000*g*, 5 min) and filtering through a PTFE syringe filter (0.22 μm pore size, Porefil) to remove any particulate matter, the clarified supernatant was loaded onto an activated Strata solid phase extraction-C18-E cartridge (55 μm, 70 Å, 500 mg/6 ml) (Phenomenex). The combined fractions of muropeptides eluted by stepwise addition of H_2_O (1 CV), 20% MeOH (2 CV), and 40% MeOH (2 CV) were evaporated, lyophilized, and resuspended in water (100 μl) for LC-MS/MS analysis, using similar condition as described above for muropeptide analysis. The identities of the anhydro-muropeptides were confirmed by MS/MS fragmentations.

### Recombinant protein overexpression and purification

For LTs, *E. coli* MltA and *MltB* genes lacking the respective signal peptide were amplified from *E. coli* K12 genomic DNA as the template. MltA[24-365] and MltB [22-361] were then cloned into pET-21a(+) vector between NheI and XhoI site *via* Gibson assembly. The sequences of plasmids, pET21_MltA[24-365]-6H and pET21_MltB[22-361]-6H were confirmed (Biobasic Sequencing). For lysozyme inhibitors, *Ivy* and *MliC* genes lacking the respective signal peptide were amplified from *E. coli* K12 genomic DNA. Ivy[29-157] and MliC[16-107] were cloned into pET-21a(+) vector between NheI and XhoI sites *via* Gibson assembly. The sequences of plasmids, pET21a(+)_Ivy[29-157]-6H and pET21a(+)_MliC[16-107]-6H were confirmed (Biobasic Sequencing). The plasmids were introduced into *E. coli* BL21 (DE3) for protein overexpression.

The procedures for MltA[24-365]-6H and MltB[22-261]-6H protein overexpression and purification were performed accordingly to previously described protocols (23). For lysozyme inhibitors, *E. coli* BL21 (DE3) cultures (LB Amp100) harboring the respective plasmids were used to inoculate 1 L of LB medium (1: 100) supplemented with 100 μg/ml Amp at 37 °C and grown to O.D._600_ ∼0.4 to 0.5 with shaking. For overexpression of Ivy[29-157]-6H, 1 mM IPTG was added and the culture was further grown at 37 °C for 3 h with shaking at 200 rpm; for overexpression of MliC[16-107]-6H, the culture was cooled to 18 °C before induction with 1 mM IPTG for additional 18 h with shaking at 200 rpm. Cells were harvested by centrifugation (4500*g*, 20 min, 4 °C) and resuspended with 30 ml ice-cold lysis buffer (20 mM Tris–HCl, pH 8.0, 150 mM NaCl, 0.1% Triton-X100, 1 mM PMSF, DNase 5 μg/ml, RNase 10 μg/ml) before sonication (30% amplitude, 10s on, 10s off, 20 min) for cell lysis. Total cell lysates were pelleted by centrifugation (13,000*g*, 20 min, 4 °C) to remove cell debris. The clarified lysate was incubated with 1.5 ml Ni-NTA resin (Qiagen) and rocked at 4 °C for 1 h. After subjecting the resins with wash buffer (100 mM sodium phosphate, pH 8.0, 40 mM imidazole), bound protein was eluted with 10 ml of elution buffer (100 mM sodium phosphate, pH 8.0, 300 mM imidazole). Eluted fractions containing Ivy-6H/MliC-6H were concentrated to < 1 ml using a 10 kDa MWCO Amicon Ultra Centrifuge Filter Device (Merck Millipore), aliquoted, and stored at −80 °C prior to use. SDS-PAGE (15% resolving ad 4% stacking gel) (Bio-Rad) was used to assess protein purity and molecular weight.

### *In vitro* analysis of LT activity and inhibition

For time-course analysis, 50 mg/ml of the *E. coli* sacculi suspension (100 ml) was treated with recombinant MltA-6H or MltB-6H (500 nM, final) in 1× PBS buffer. The reaction was incubated at 37 °C with shaking for a different amount of time, after which samples were quenched by heating at 95 °C for 10 min. The supernatant containing soluble muropeptides was collected by centrifugation (15,000*g*, 20 min) for LC-MS analyses as described above. For analysis of the inhibitory effects of lysozyme inhibitors, recombinant Ivy-6H or MliC-6H proteins were added at indicated stoichiometric ratios to each LT reaction for incubation of 20 min at 37 °C with shaking prior to heat inactivation (95 °C, 10 min). The supernatant containing the soluble muropeptides was subjected to LC-MS/MS analysis as described above.

### Quantitative proteomics analysis

Overnight culture of pelB-MltB–expressing *E. coli* MG1655 was refreshed (1:100) in LB broth supplemented with Cm30 and grown at 37 °C with shaking at 200 rpm. For the uninduced sample (Sample UI), the culture (20 ml) was harvested at O.D.600 ∼0.8. For the restored sample (MltB LA), L-Ara (0.1%, final) was added when the culture was at O.D.600 ∼0.6. The culture underwent characteristic lysis followed by restoration and was harvested when the culture restored growth at O.D.600 ∼0.8. For the mutant sample (MltB-Mut LA), the pelB-MltB-E162Q–expressing *E. coli* MG1655 was used instead. For consistency in sample preparation, L-Ara (0.1%, final) was added to the culture at O.D._600_ ∼0.6. The mutant sample did not exhibit any lysis; upon reaching saturation, it was refreshed 2-fold (LB supplemented with Cam30 and 0.1% L-Ara) upon reaching saturation (O.D.600 ∼0.8) two times. The sample was harvested at final O.D._600_ ∼0.8 at the other ones. Cell pellets were collected by centrifugation of harvest sample (5000 *g*, 20 min), followed by resuspension in 1 ml lysis buffer (20 mM sodium phosphate pH 7.5, 140 mM NaCl, and 1 mM PMSF) and sonication (10s on, 10s off, 30% amplitude; 5 times). The total lysate was centrifugation (13,000 g, 30 min) to remove cell debris and concentrated by 10 kDa Amicon (Merck) to ∼ 20 mg/ml for iTRAQ proteomics analysis at Protein and Proteomics Centre at National University of Singapore (NUS).

Identification and quantification of proteins were performed by the Protein and Proteomics Centre, NUS. Briefly, proteins were identified by matching the MS data against a combined database with ProteinPilot (SCIEX, v. 5.02). The combined database comprises a pan reference proteome from *E. coli* strain K-12 (UniProt UP000000625, 2020 July release, 4391 entries) (71, 72) and common contaminant proteins (cRAP) (73). MltB LA was compared against both Sample UI and MltB-Mut LA. Student’s *t* test was performed for both comparisons. Proteins were considered differentially in both comparisons (*e.g.*, significantly upregulated in both comparisons). Gene ontology analysis of DE proteins was conducted using the ShinyGO web app (version 0.76.3, http://bioinformatics.sdstate.edu/go/) (74) with default settings and background proteins provided. Proteins belonging to the Rcs pathway were annotated with reference to literature (38).

### Colanic acid extraction and quantification

Colanic acid was extracted and quantified following the literature protocol (75) with modifications. In brief, the uninduced or restored cell pellets of the pelB-MltA–expressing *E. coli* MG1655 (25 ml) were suspended in 10 ml 1× PBS, boiled at 100 °C for 15 min, and cooled down. To the supernatant after centrifugation (2400*g*, 5 min), cold EtOH (30 ml) was added and incubated for at least 5 h for precipitation of colanic acid. The colanic acid precipitates were collected by centrifugation (2400 g, 5 min) and then resuspended in 1 ml D.I. water.

Colanic acid content was quantified by measuring the concentration of L-fucose present ([L-fucose]). Four hundred fifty microliters of colanic acid resuspension were added with 1 ml H2SO4 (87%, v/v) on ice prior to heating at 100 °C for 20 min. To 1 ml of the resulting solution, l-cysteine hydrochloride (1 M, 30 μl) was added. A_396_ and A_427_ was measured before and after l-cysteine addition (NanoDrop One Spectrophotometer, Thermo Fisher Scientific) and ΔA was calculated, where ΔA = (post-A_396_ - pre-A_396_) - (post-A_427_ - pre-A_427_). [l-fucose] was calculated using a linear calibration curve ([l-fucose] = 0 to 100 μg/ml, r^2^ = 0.99).

### Quantitative PCR analysis

The total RNA of the indicated sample was isolated with Miniprep Kit (NEB) and reverse transcribed into complementary DNA (cDNA) with oligo (dT) primers with First Strand cDNA Synthesis Kit (NEB) according to standard procedures. Quantitative PCR analyses were performed in an Eco Real-time PCR system (Illumina). With cDNA served as the template, each reaction was set up using the SYBR Green 2× qPCR MasterMix (YouSeq Tetra) according to the manufacturer’s instructions. Initial denaturation at 95 °C for 10  min was followed by 40 cycles, each cycle consisting of 95 °C for 10 s, the primer-specific annealing temperature for 5 s, and elongation at 60 °C for 10 s. The putative patatin-like phospholipase (rssA) was used as a housekeeping gene standard (sense 5′ AGG ACT CAT GGC ACC TGT TG3’; antisense 5′ GCA GGT CAA CCG CTA TCA CA3′). The primers designed for other LTs based on the *E. coli* MG1655 genome were listed in Table S3. At the end of each run, melting curve profiles were achieved by cooling the sample to 55 °C for 15 s and then steadily increasing to 95 °C with continuous measurement of fluorescence to confirm the amplification of specific transcripts. The specificity of designed primers was verified by subjecting the cDNA synthesized to electrophoresis on a 2% agarose gel and visualization by SYBR staining (Thermo Fisher Scientific). Relative mRNA expression was calculated using the 2^–ΔΔCt^ method with reference to the rssA gene expression.

### Efficiency-of-plating assay

Efficiency-of-plating analyses were performed on LB agar plates containing appropriate antibiotics with or without 0.1% L-Ara as indicated. For colony forming units counting, bacterial culture before and after the addition of L-Ara for LT-overexpressing strain was harvested, serially diluted, and spread (50 μl) on agar plates. The plates were incubated at 30 °C for 48 h before counting the viable bacteria colonies. For validation of the quantitative proteomics results of Rcs upregulation in LT-overexpressing strain, overnight *E. coli* culture was normalized by diluting to an O.D.600 of 0.2 and serially diluted before manually spotted (2 μl) onto agar plates. The plates were dried and incubated overnight at 37 °C prior to imaging. X-gal LB plates were used for the analysis of Rcs phosphorelay activation in DH300 rprA-lacZ reporter strain. Briefly, a stock solution of X-gal (40 mg/ml) was reconstituted freshly in dimethylformamide before spreading onto plates (400 μg/ml, final). Plate images were taken by the Amersham ImageQuant 800 (GE Healthcare).

### MIC determination

MIC determination was performed using the broth microdilution method. Briefly, a single colony of *E. coli* MG1655 or MG1655_Δ*rcsf* harboring the pelB-MltA plasmid was inoculated in LB with Cm30 for overnight growth at 37 °C with shaking at 200 rpm. The saturated culture was diluted with fresh LB supplemented with Cm30 to O.D._600_ ∼0.3, and 100 μl was added to a flat-bottom 96-well plate containing 2-fold serial dilutions of respective antibiotics, such as carbenicillin, oxacillin, penicillin G sodium, colistin sulfate (Sigma Aldrich), and kanamycin monosulfate (GOLDBIO) supplemented with or without L-Ara (0.1%, final). Plates were covered and statically incubated for 18  h at 37 °C before taking O.D._600_ reading. The concentration of the drug at which there is no bacterial growth (O.D._600_ < 0.1) was observed and recorded as the MIC.

### Statistical analysis

Data were analyzed using GraphPad Prism, version 9.0, software (GraphPad Inc.). All values are reported as mean ± SD, and the difference between group means was evaluated using One-way ANOVA or Student’s *t* test as indicated in the figure legends. Significance was established at *p* values ≤ 0.05 for statistical analysis.

## Data availability

All data are included in the article.

## Supporting information

This article contains supporting information including references (26, 39, 76).

## Conflict of interest

The authors declare that they have no conflicts of interest with the contents of this article.
