# Supplementary material for: Escherichia coli has robust regulatory mechanisms against elevated peptidoglycan cleavage by lytic transglycosylases
Source: J Biol Chem. 2023 Mar 16;299(4):104615. doi: 10.1016/j.jbc.2023.104615 (PMC10139938; doi:10.1016/j.jbc.2023.104615)
Supplement: Supporting Information [file mmc1.docx]

**Supporting Information**

*Escherichia coli* has robust regulatory mechanisms against the elevated peptidoglycan cleavage by lytic transglycosylase

Yaquan Liang^1^, Yilin Zhao^1^, Jeric Kwan^1^, Yue Wang^2^, Yuan Qiao^1^*

^1^School of Chemistry, Chemical Engineering and Biotechnology, Nanyang Technological University, Singapore 637371

^2^A*STAR Infectious Diseases Labs, Singapore 138648

^*^ For correspondence, Yuan Qiao, [yuanqiao@ntu.edu.sg](mailto:yuanqiao@ntu.edu.sg)

**Running Title:** *Keeping the aberrant peptidoglycan cleavage in check*

Includes:

**Supplementary Figures S1 to S6**

**Supplementary Tables S1 to S3**


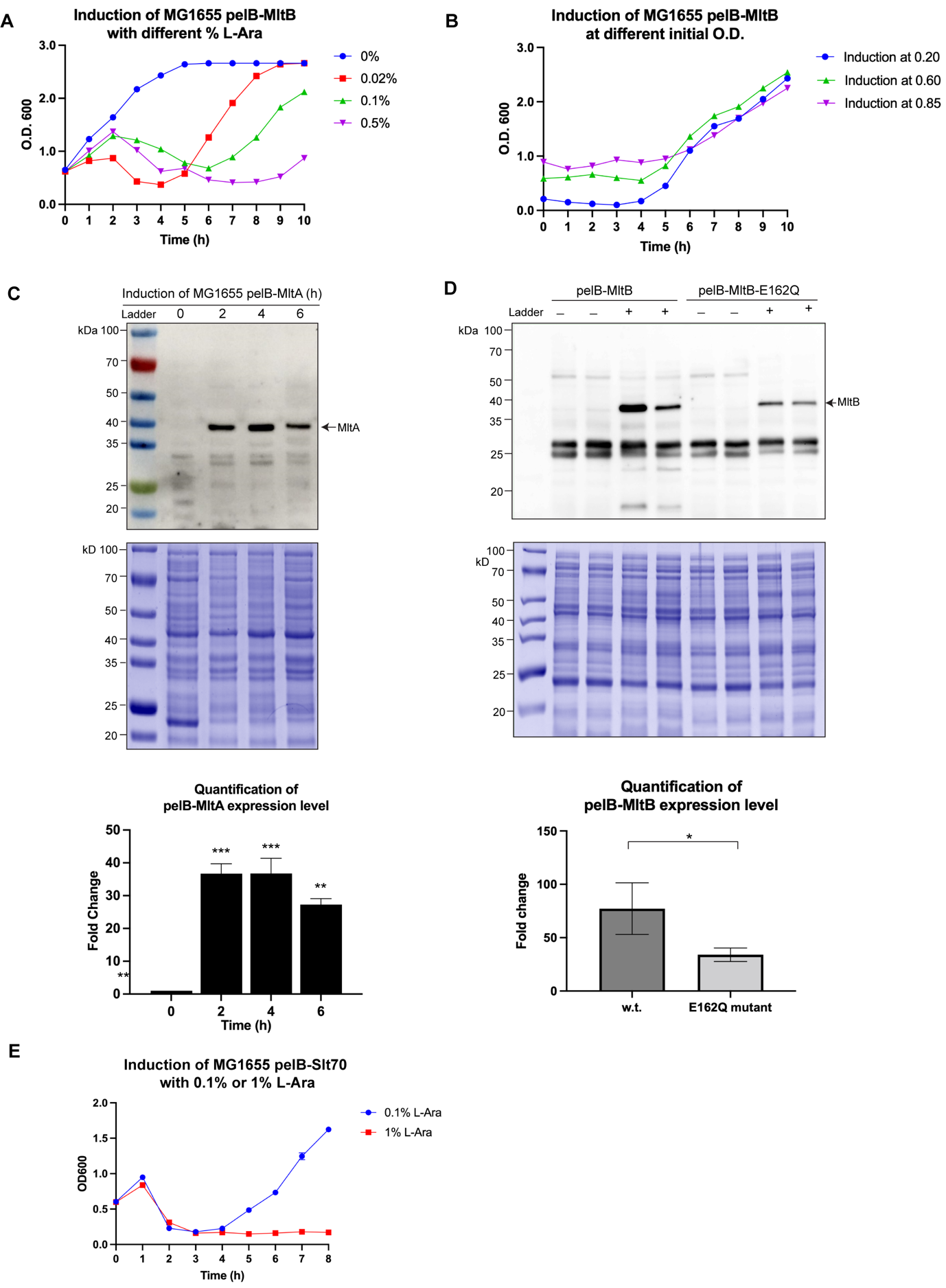


**Figure S1. Growth trend and Western blot analyses of *E. coli* MG1655 harboring pelB-MltA/B. (A)** Growth curves of *E. coli* MG1655-pelB-MltB with the addition of the different amounts of L-arabinose (L-Ara) inducer (0%, 0.02%, 0.1% and 0.5%) at O.D._600_ ~0.6. All cultures show characteristic lysis (*i.e.,* decrease in OD) followed by restoration (*i.e.,* increase in OD) under different L-Ara used. **(B)** Growth curves of *E. coli* MG1655-pelB-MltB when induced with 0.1% L-Ara at different initial O.D._600_. All cultures were able to restore growth. **(C)** Anti-His_6_ immunoblotting of cell lysate from *E. coli* MG1655-pelB-MltA at indicated time points upon L-Ara induction shows overexpression of MltA. **(D)** Anti-His immunoblotting of cell lysates from *E.coli* MG1655 pelB-MltB and pelB-MltB-E162Q (active site mutant) treated without or with L-Ara indicates overexpression of both wildtype and mutant MltB, respectively. **(E)** Growth curve of *E. coli* MG1655-pelB-Slt70 with the addition of 0.1% or 1% L-Ara at O.D._600_ ~0.6. Quantification of the band intensity was performed using ImageJ with three independent biological replicates. Statistical analysis of the blots was performed using ordinary one-way ANOVA, results are presented as mean + standard deviation (SD). **p* <0.05, ** *p* <0.01, ****p*<0.001.


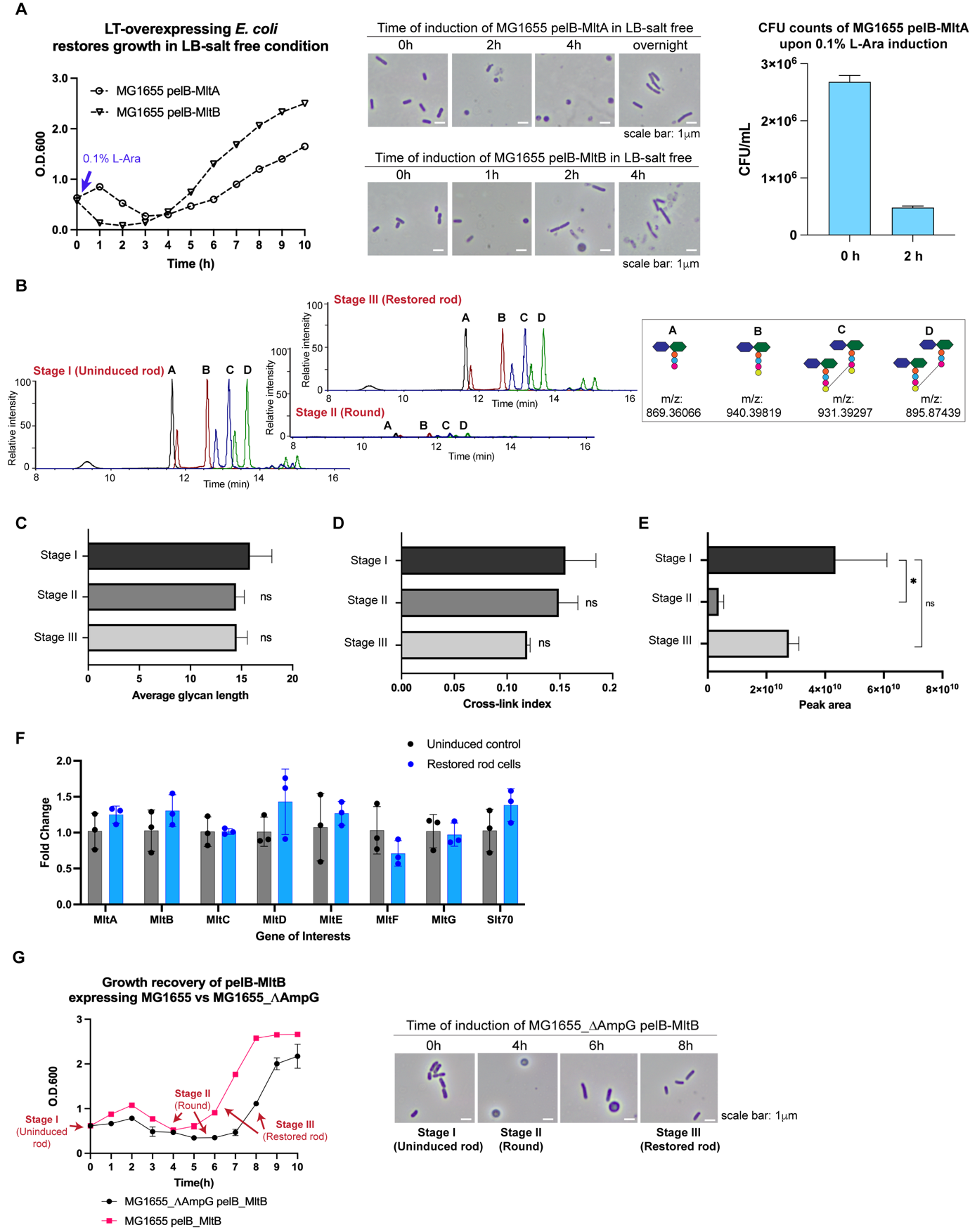


**Figure S2. Peptidoglycan profile and soluble anhydro-muropeptide analysis of LT-overexpressing *E. coli*. (A**) Growth curves of *E. coli* MG1655-pelB-MltA/B under LB-salt free condition upon L-Ara induction. Both strains show the characteristic trend of lysis followed by growth restoration, the representative morphological images at various time points are shown on the right. Scale bar: 1μm. Cell lysis was confirmed by CFU counting of viable cells in MG1655-pelB-MltA upon induction of L-Ara. (**B)** LC-MS analysis of peptidoglycan profiles of *E. coli* MG1655-pelB-MltB harvested at three morphological stages (i.e. uninduced, round and restored). Extracted ion chromatograms (EIC) of four muropeptides (A-D) were shown. (**C-E)**, Analyses of spectra in **B** reveal no significant changes in terms of average glycan length and cross-link index across three samples, while the stage II round cells display significantly lower total muropeptide abundance consistent with the weakened peptidoglycan layer in these cells. **(F)** RT-qPCR analysis of gene expressions of the endogenous LTs in *E. coli* in uninduced or in restored rod cells. (**G**) Growth curves of the pelB-MltB expressing MG1655 and MG1655_ΔAmpG cells show similar trends upon L-Ara induction. The representative morphological images of pelB-MltB expressing MG1655_ΔAmpG at the three stages (I, II and III) are shown on the right. Scale bar: 1μm. Statistical analysis of the results was performed using ordinary one-way ANOVA with graphs presented as mean + standard deviation (SD). **p* <0.05, ***,* ns = not significant.


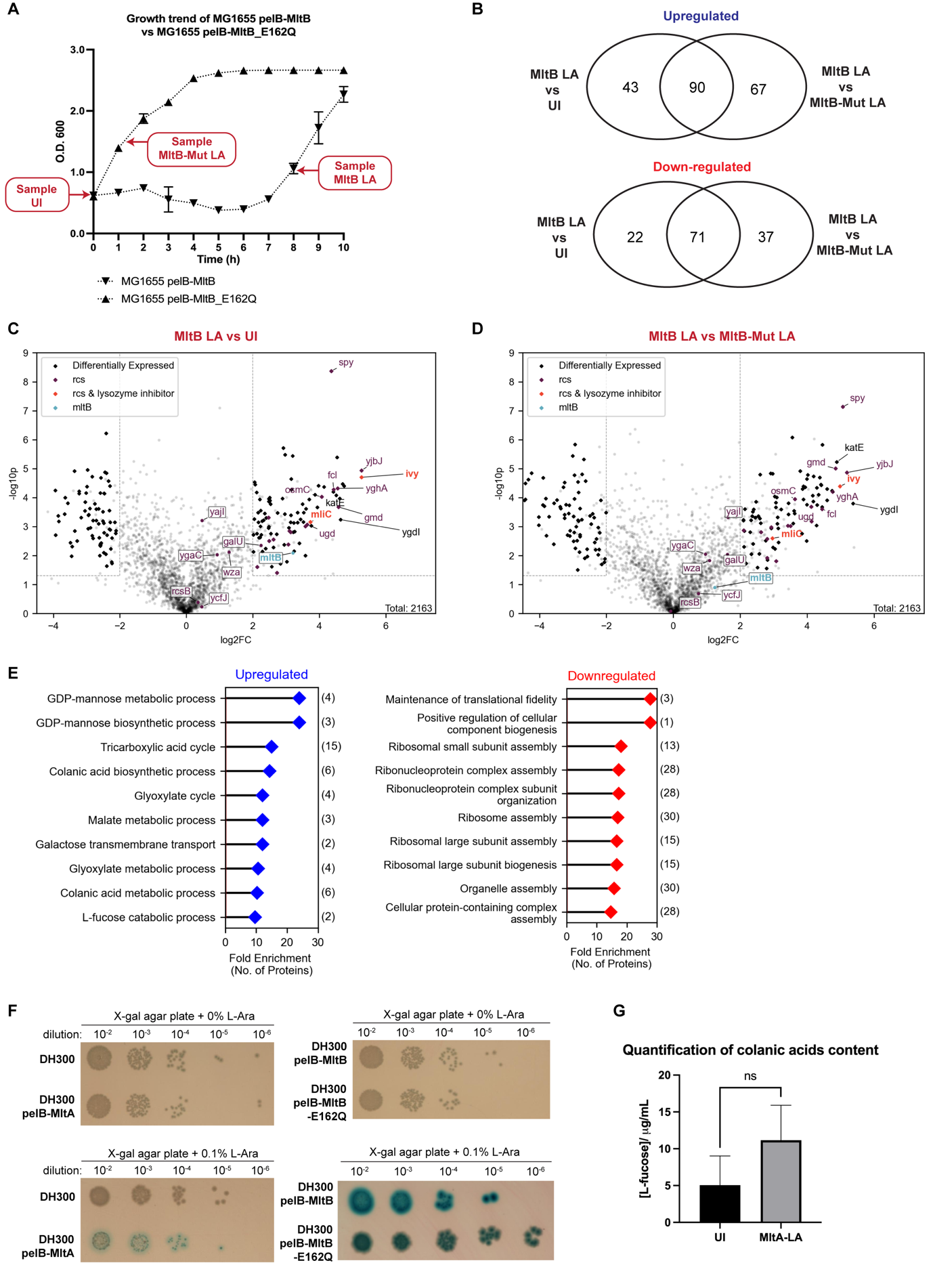


**Figure S3. Elevated periplasmic LT activity upregulates Rcs stress regulon. (A)** Sample harvested at indicated time points for quantitative proteomics analysis. Sample UI: uninduced; sample MltB LA: MG1655 pelB-MltB treated with 0.1% L-Ara; sample MltB-Mut LA: MG1655 pelB-MltB-E162Q treated with 0.1% L-Ara. All samples were harvested at similar OD ~0.8 with biological duplicates for iTRAQ analysis. **(B)** Venn diagrams show the set of differentially expressed proteins (either upregulated or downregulated, |log2FC| > 2 and p-value < 0.05) that are specific to sample MltB LA. **(C-D)** Volcano plots of the differentially expressed proteins in MltB LA reveal hits in the Rcs regulon (colored in purple). Note: For ease of comparing two plots, the volcano plot in **Figure S3C** is reused from **Figure 4A**. **(E)** Gene Ontology pathway analysis of the differentially expressed hits. **(F)** Validation of Rcs upregulation upon LT elevation using the DH300 *rprA-lacZ* reporter strain harboring respective pelB-MltA/B plasmid. For the bottom left panel, the plate contains 400 μg/mL X-gal; for the bottom right panel, the plate contains 2 mg/mL X-gal for better distinction between the pelB-MltB and pelB-MltB-E162Q-possessing strains. Note: For ease of comparison, the agar plate images in the left panel of **Figure S3F** are reused from **Figure 4B.** **(G)** Validation of colanic acid upregulation in sample MltA LA compared to UI. Results are average of three biological replicated and presented as mean + standard deviation (SD) using student’s *t*-test. ** *p* <0.01,


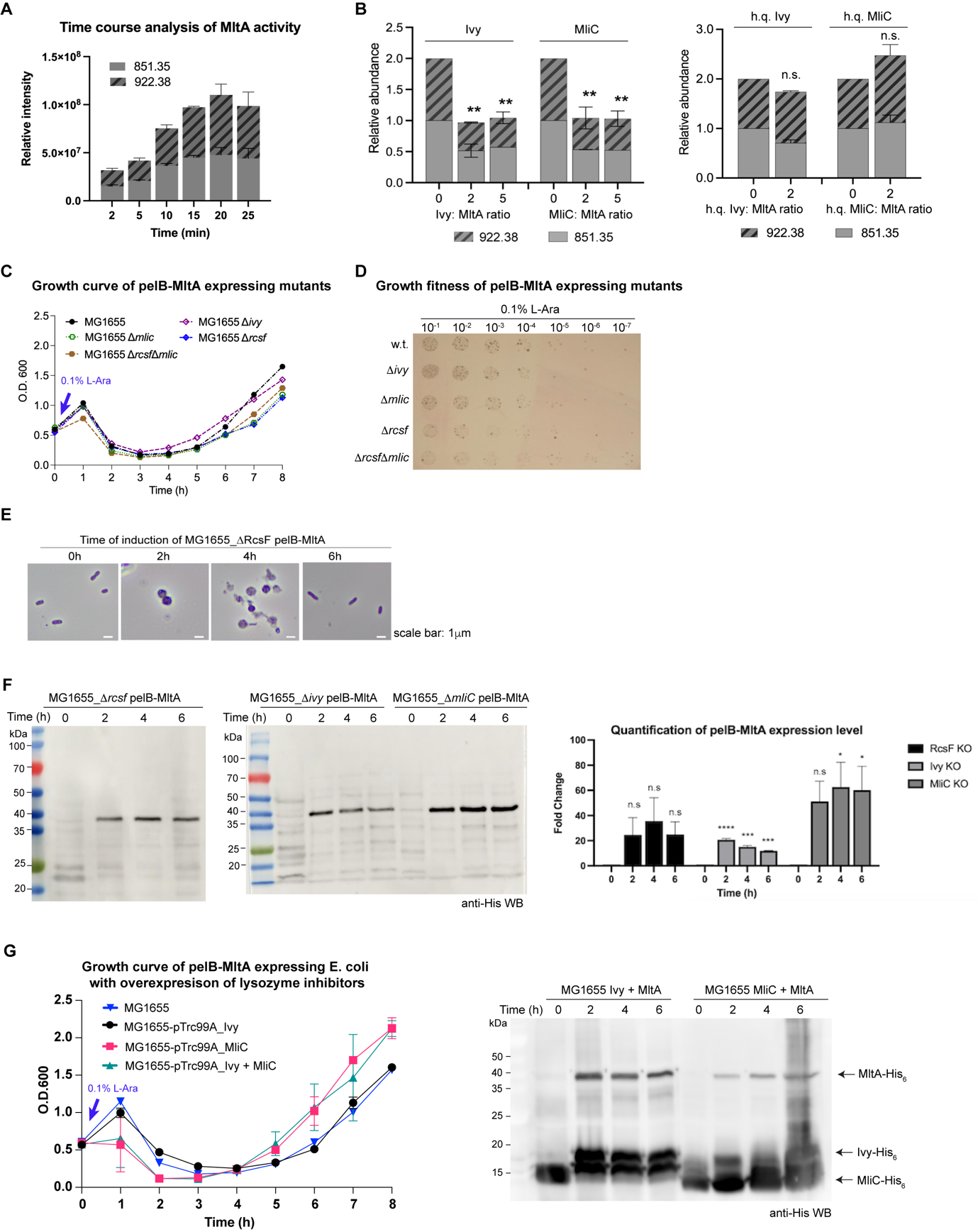


**Figure S4. Evaluation of lysozyme inhibitors Ivy and MliC against *E. coli* MltA. (A)** LC-MS-based time-course study of the activity of recombinant *E. coli* MltA *in vitro.* The amounts of anhydro-muropeptides (m/z: 851.35, 922.38) produced by non-hydrolytic cleavage of *E. coli* sacculi by MltA are quantified, as a readout of enzymatic activity. **(B)** The addition of excess lysozyme inhibitor, Ivy or MliC, partially inhibits the activity of MltA, whereas heat-quenched Ivy or MliC exhibits no inhibitory activity at the stoichiometric excess used. **(C-D)** Growth trend analyses of pelB-MltA expressing *E. coli* MG1655 and different mutants in liquid culture or agar plate upon L-ara induction. MG1655_Δ*ivy* and Δ*mliC* strains harboring pelB-MltA show similar restoration as the wildtype, while MG1655_Δ*rcsf* exhibits more pronounced delays in growth restoration. **(E)** Morphological changes of pelB-MltA-expressing MG1655_Δ*rcsf* at different time points upon induction. Scale bar: 1 μm. **(F)** Anti-His immunoblotting of cell lysates from pelB-MltA possessing *E.coli* MG1655 mutants shows overexpression of MltA upon induction. **(G)** Growth curves of pelB-MltA expressing *E. coli* MG1655 with overexpression of lysozyme inhibitors (Ivy or MliC or both). Immunoblots confirm the overexpression of Ivy, MliC, and MltA upon induction. Quantification of band intensity was performed using ImageJ Software with at least three independent biological replicates. Statistical analysis of the blots was performed using ordinary one-way ANOVA, results are presented as mean + standard deviation (SD). **p* <0.05, ** *p* <0.01.


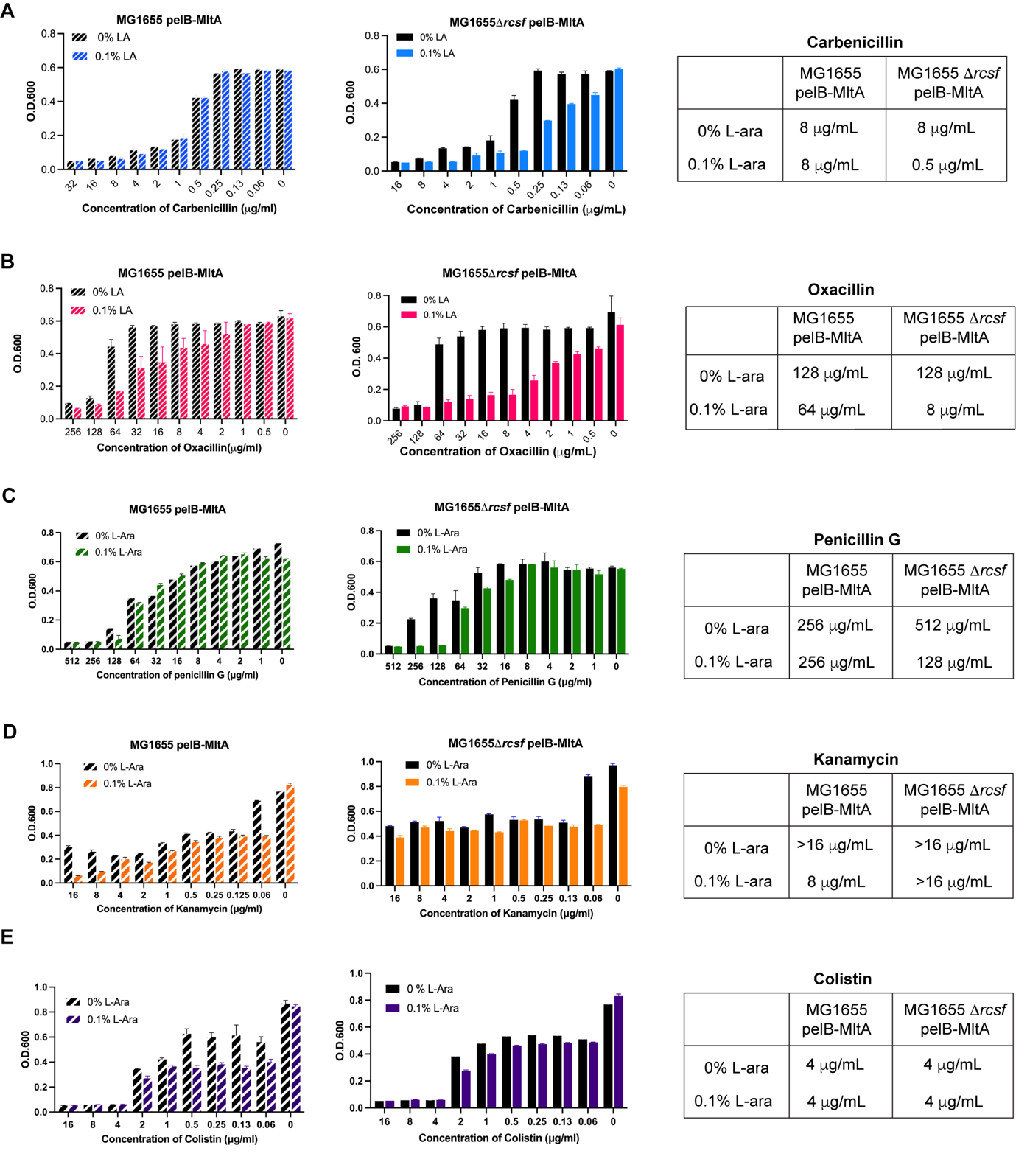


**Figure S5. Elevated LT activity potentiates beta-lactam antibiotics against *E. coli* MG1655_Δ*rcsf* background. (A-E)** Endpoint O.D_.600_ measurements of pelB-MltA possessing MG1655 and MG1655_Δ*rcsf* strains with or without L-Ara inducer treated with a serial dilution of different antibiotics. The induction of pelB-MltA (with 0.1% L-ara) in MG1655_Δ*rcsf* background specifically sensitizes beta-lactam drugs such as carbenicillin, oxacillin and penicillin G **(A-C)**, but no effects for non-PG targeting antibiotics such as kanamycin and colistin sulfate salt **(D-E)**.

**Table S1. Bacterial strains used in the study**

| **Strains** | **Description/Genotype** | **Source/Reference** |
| --- | --- | --- |
| MG1655 | F^−^ λ^−^ *ilvG rfb-50 rph-1* | Lab collection |
| DH5α | *E. coli F’/hsd*R17*, recA1, gyrA* | Lab collection |
| BL21(DE3) | *E. coli B dcm ompT hsdS*(r_B_^-^m_B_^-^) *gal* | Lab collection |
| DH300 | *rprA*-*lac*Z MG1655 *Δ*(*argF-lac*)*U169* | Majdalani *et al*., 2002 (39) (Gift from Jean-François Collet lab, de Duve Institute) |
| MG1655 *ΔampG* | MG1655 *ΔampG::kan* | This study |
| MG1655 *Δrcsf* | P1-transduced from JW0192 and JW4005, Kan cassette removed | This study |
| MG1655 *Δivy* | P1-transduced from JW0192 and JW4005, Kan cassette removed | This study |
| MG1655 *ΔmliC* | P1-transduced from JW0192 and JW4005, Kan cassette removed | This study |
| MG1655 *Δrcsf ΔmliC* | P1-transduced from JW0192 and JW4005, Kan cassette removed | This study |
| JW0192 | BW25113 *Δrcsf::kan* | Kieo collection  (Gift from Jean-François Collet lab, de Duve Institute) |
| JW0210 | BW25113 *Δivy::kan* | Kieo collection |
| JW4005 | BW25113 *ΔmliC::kan* | Kieo collection |

**Table S2. Plasmids used in this study**

| **Plasmid** | **Features/Descriptions** | **Source/Reference** | **Primers** |
| --- | --- | --- | --- |
| pET-21a(+) | IPTG-regulated T7promoter; Amp^r^ | Novagen |  |
| pBAD33 | Arabinose regulation; Cm^r^ | Guzman *et al.,* 1995 (26) |  |
| pBAD33-RBS-MltA[1-365]-6H | For inducible expression of full length MltA in *E. coli* MG1655; used for growth phenotype analysis | This study | EcMltAfl_01, EcMltAfl_02 |
| pBAD33-RBS-MltB[1-361]-6H | For inducible expression of full length MltB in *E. coli* MG1655; used for growth phenotype analysis | This study | EcMltBfl_01, EcMltBfl_02 |
| pBAD33-RBS-Slt70[1-648]-6H | For inducible expression of full length slt70 in *E. coli* MG1655; used for growth phenotype analysis | This Study | EcSlt70fl_01, EcSlt70fl_02 |
| pBAD33-RBS-pelB-MltA[24-365]-6H | For inducible expression of soluble domain of MltA[25-365] in *E. coli* MG1655 periplasm; used for growth phenotype analysis | This study | EcMltA24-001  EcMltA24-002  EcMltA/B-003  EcMltA/B-004 |
| pBAD33-RBS-pelB-MltB[22-361]-6H | For inducible expression of soluble domain of MltB[25-361] in *E. coli* MG1655 (or mutants) periplasm; used for growth phenotype analysis | This study | EcMltB22-001  EcMltB22-002  EcMltA/B-003  EcMltA/B-004 |
| pBAD33-RBS-pelB-Slt70[28-645]-6H | For inducible expression of soluble domain of Slt70[28-645] in *E. coli* MG1655 (or mutants) periplasm; used for growth phenotype analysis | This study | EcSlt70-001  EcSlt70-002  EcMltA/B-003  EcMltA/B-004 |
| pBAD33-RBS-pelB-MltB[22-361]-E162Q-6H | For inducible expression of soluble domain of catalytic mutant of MltB[22-361]_E162Q in *E. coli* MG1655 periplasm; used for growth phenotype analysis | This study | EcMltB_E162Q |
| pTr99a-RBS-ivy-6H | For inducible expression of ivy | This study | IvyRE_001 |
| pTr99a-RBS-mliC-6H | For inducible expression of mliC | This Study | mliCRE_001 |
| pET21a(+)_MltA[24-365]-6H | Protein overexpression and purification | This study | EcMltApET |
| pET21a(+)_MltB[22-361]-6H | Protein overexpression and purification | This study | EcMltBpET |
| pET21a(+)_Ivy[29-157]-6H | Protein overexpression and purification | This study | EcIvypET |
| pET21a(+)_MliC[16-107]-6H | Protein overexpression and purification | This study | EcMliCpET |
| pKD13 | Construction of gene knockout;  *Δrcsf, Δivy, ΔmliC, ΔmliCΔrcsf* | Baba *et al*., 2006 (76) (Gift from Chng lab, NUS) | EcIvy_001  EcMliC_001  EcRcsf_001 |
| pCP20 | Construction of gene knockout;  *Δrcsf, Δivy, ΔmliC, ΔmliCΔrcsf* | Baba *et al.,* 2006 (76) (Gift from Chng lab, NUS) | EcIvy_001  EcMliC_001  EcRcsf_001 |
| pKD46 | Construction of gene knockout; Kan^r^  *ΔampG* | Baba *et al.,* 2006 (76) (Gift from Chng lab, NUS) |  |

Abbreviations: Amp^r^, Ampicillin resistance; Cm^r^, chloramphenicol resistance, Kan^r^, kanamycin resistance.

**Table S3. Primers used for recombinant protein expression, cloning, and qPCR.**

| **Primer Name** | **Sequence (5’ to 3’)** (template specific binding region is indicated by underlines) |
| --- | --- |
| EcMltBpET | FWD: ctttaagaaggagatatacatatggATGAAGCCAAAACCTACTGAGACTG  REV: tggtggtggtggtgcTGTACTCGCGCCAGC |
| EcMltApET | FWD: ctttaagaaggagatatacatatggATGAAACCAACCGATCGCGG  REV: tggtggtggtggtgcCAGCCGCTAAAGACGTTACC |
| EcIvypET | FWD: ACTTTAAGAAGGAGATATACATATGGcgCAGGATGATTTAACCATTAGCAG  REV: GGTGGTGGTGCTCGAaTTTAAAATTAAAGCCATCCgga |
| EcMliCpET | FWD: ACTTTAAGAAGGAGATATACATATGGgcTGTAGCGCCTTTAATCAGC  REV: GGTGGTGGTGCTCGAaACGCTGTGGATTTTGTAACT |
| EcAmpG_KO | FWD: CCCTCTGGCCCGGTGCAAGCCGGGCCTGTAGACGCCCATGATTCCGGGGATCCGTCGACC  REV:  ACAGCTAAATAATATTTACAGATTACGTCAGATGCGTTTTTGTAGGCTGGAGCTGCTTCG |
| EcMltAfl_01 | FWD: **AAAGAGGAGAAA**TACTAGATGAAAGGACGTTGGGTAAAGT (RBS is bolded)  REV: TCAgtgatggtgatggtgatgGCCGCTAAAGACGTTACCT |
| EcMltAfl_02 | FWD: atcaccatcaccatcacTGAggatcctctagagtcgacct  REV: ATCTAGTATTTCTCCTCTTTgctcgaattcgctagcccaa |
| EcMltBfl_01 | FWD: **AAAGAGGAGAAA**TACTAGATGTTCAAGCGTCGTTATGTAAC (RBS is bolded)  REV: TCAgtgatggtgatggtgatgCTGTACTCGCGCCAGC |
| EcMltBfl_02 | FWD: ttgggctagcgaattcgagcAAAGAGGAGAAATACTAGATGAAAG  REV: aggtcgactctagaggatccTCAgtgatggtgatggtgat |
| EcSlt70fl_01 | FWD: **AAAGAGGAGAAA**TACTAGATGTTCAAGCGTCGTTATGTAAC (RBS is bolded)  REV: TCAgtgatggtgatggtgatgCTGTACTCGCGCCAGC |
| EcSlt70fl_02 | FWD: gcAAAGAGGAGAAATACTAGATGGAAAAAGCCAAACAAGTTACC  REV: CAgtgatggtgatggtgatgGTAACGACGTCCCCATTCC |
| EcMltA24_001 | FWD: atcaccatcaccatcacTGAGATCCGGCTGCTAACAAAGCCCGA  REV: TGCTGTCCGCGATCGGTTGGGGCCATCGCCGGCTGGGCAG |
| EcMltA24_002 | FWD: CTGCCCAGCCGGCGATGGCCCCAACCGATCGCGGACAGCA  REV: GCTTTGTTAGCAGCCGGATCTCAgtgatggtgatggtgatgGC |
| EcMltB22_001 | FWD: atcaccatcaccatcacTGAGATCCGGCTGCTAACAAAGCCCGA  REV: GTCTCAGTAGGTTTTGGCTTGGCCATCGCCGGCTGGGCAG |
| EcMltB22_002 | FWD: CTGCCCAGCCGGCGATGGCCAAGCCAAAACCTACTGAGAC  REV: GCTTTGTTAGCAGCCGGATCTCAgtgatggtgatggtgat |
| EcSlt70_001 | FWD: ATCACCATCACCATCACTGAGGATCCTCTAGAGTCGACCT  REV: TGCTGTCCGCGATCGGTTGGGGCCATCGCCGGCTGGGCAG |
| EcSlt70_002 | FWD: ATCGACGAATTCGGACTCACTGGATGAGCAGCG  REV: ACTCGACTCGAGGTAACGACGTCCCCATTCCG |
| EcMltA/B-003 | FWD: atcaccatcaccatcacTGAggatcctctagagtcgacct  REV: GTCGGCAGCAGGTATTTCATCTAGTATTTCTCCTCTTTgctc |
| EcMltA/B-004 | FWD: gcAAAGAGGAGAAATACTAGATGAAATACCTGCTGCCGAC  REV: aggtcgactctagaggatccTCAgtgatggtgatggtgat |
| EcMltB_E162Q | FWD: GGATTATCGGCGTTcaaACCCGCTGGGG  REV: CCCCAGCGGGTttgAACGCCGATAATCC |
| IvyRE_001 | FWD: atgcatgaattcATGGGCAGGATAAGCTCGGGAGG  REV: tgaaagcttTTAgtgatggtgatggtgatgTTTAAAATTAAAGCCATCc |
| mliCRE_001 | FWD: ctagaattcATGACTATGAAAAAACTGTTAATTATCATTTTGC  REV: tataagctttcagtgatggtgatggtgatgACGCTGTGGATTTTGT |
| EcIvy_001 | FWD: GATTTGCAAATTATCGTGTTATCGCCAGGCTTTAGGAGGTTAATAACATG  REV: CCGAAAGGCTCCGTTTCTTTATCCGCTAATTATTTAAAATTAAAGCCATC |
| EcMliC_001 | FWD: CGCTATTGTGCGCCGCCCCTGGAAAAATCTCAACGCTGTGGATTTTGTAA  REV: CGTTAGGGAGGGCGTATTGCCCTCCAGACCAGGAAAGTCTTCGGGATATG |
| EcRcsf_001 | FWD: AACGCCTATTTGCTCGAACTGGAAACTGCTCATTTCGCCGTAATGTTAAG  REV: GCTCCTGATTCAATATTGACGTTTTGATCATACATTGAGGAAATACTATG |
| qMltA | FWD: CACCAGAGAGAAAGGCTGGG  REV: TCTTATGGGCACGGTTGTGG |
| qMltB | FWD: TGCTGGGCATTCGGGTTATT  REV: AGACTGATACGACCACCGGA |
| qMltC | FWD: AAAGAGGTGGTGATCGCTGG  REV: CCGGCGATGGTTTCGATAGT |
| qMltD | FWD: TTTCATCGGTCGTTGGCAGA  REV: GAACAAAGCGCGTGGGAAAT |
| qMltE | FWD: ATTTCAATGGGGGCGGCTTA  REV: TGACACCACCAGCGCATATT |
| qMltF | FWD: TGCTGGCCGCTATTGCTTAT  REV: GAGGCTTTGCGCGGTATTTT |
| qMltG | FWD: ACTTACCGCTTTACACCGCA  REV: TAATCGCTCAGACGCATCCC |
| qSlt70 | FWD: GGCAGGCGGATTTATTGCTG  REV: CATCGGGTAGAAACCACGCT |

Abbreviations: FWD, forward primer; REV, reverse primer.

**References**

(26) Guzman, L. M., Belin, D., Carson, M. J., and Beckwith, J. (1995) Tight regulation, modulation, and high-level expression by vectors containing the arabinose PBAD promoter. *J Bacteriol*. **177**, 4121–4130

(39) Majdalani, N., Hernandez, D., and Gottesman, S. (2002) Regulation and mode of action of the second small RNA activator of RpoS translation, RprA. *Mol Microbiol*. **46**, 813–26

(76) Baba T, Ara T, Hasegawa M, Takai Y, Okumura Y, Baba M, Datsenko KA, Tomita M, Wanner BL, Mori H. (2006) Construction of Escherichia coli K-12 in-frame, single-gene knockout mutants: the Keio collection. *Mol Syst Biol.* **2**, 2006.0008.
